# Heat treatment-induced autophagy promotes breast cancer cell invasion and metastasis via TGF-*β*2-mediated epithelial-mesenchymal transitions

**DOI:** 10.7717/peerj.14640

**Published:** 2023-01-12

**Authors:** Zhennan Li, Cheng Lu, Fengliang Wang, Haowei Guo, Zhipeng Wang, Hong Yin, Jian Li

**Affiliations:** 1 Department of Breast Surgery, Women’s Hospital of Nanjing Medical University, Nanjing Maternity and Child Health Care Hospital, Nanjing, Jiangsu, China; 2 Department of Pulmonary Medicine, Affiliated Hospital of Jiangsu University, Zhenjiang, Jiangsu, China

**Keywords:** Breast cancer cell, Microwave ablation, Sublethal heat treatment, Autophagy, Epithelial-mesenchymal transition, Metastasis

## Abstract

**Background:**

Insufficient thermal ablation can accelerate malignant behaviors and metastases in some solid tumors, and epithelial-mesenchymal transition (EMT) and autophagy are involved in tumor metastasis. It has been found that TGF-*β*2 which belongs to the family of transforming growth factors often associated with cancer cell invasiveness and EMT. However, whether the interactions between autophagy and TGF-*β*2 induce EMT in breast cancer (BC) cells following insufficient microwave ablation (MWA) remains unclear.

**Methods:**

BC cells were treated with sublethal heat treatment to simulate insufficient MWA, and the effects of heat treatment on the BC cell phenotypes were explored. CCK-8, colony formation, flow cytometry, Transwell, and wound healing assays were performed to evaluate the influence of sublethal heat treatment on the proliferation, apoptosis, invasion, and migration of BC cells. Western blotting, real-time quantitative PCR, immunofluorescence, and transmission electron microscopy were carried out to determine the changes in markers associated with autophagy and EMT following sublethal heat treatment.

**Results:**

Results showed that heat treatment promoted the proliferation of surviving BC cells, which was accompanied by autophagy induction. Heat treatment-induced autophagy up-regulated TGF-*β*2/Smad2 signaling and promoted EMT phenotype, thereby enhancing BC cells’ migration and invasion abilities. An increase or decrease of TGF-*β*2 expression resulted in the potentiation and suppression of autophagy, as well as the enhancement and abatement of EMT. Autophagy inhibitors facilitated apoptosis and repressed proliferation of BC cells *in vitro*, and thwarted BC cell tumor growth and pulmonary metastasis *in vivo*.

**Conclusion:**

Heat treatment-induced autophagy promoted invasion and metastasis via TGF-*β*2/Smad2-mediated EMTs. Suppressing autophagy may be a suitable strategy for overcoming the progression and metastasis of residual BC cells following insufficient MWA.

## Introduction

Breast cancer (BC) is the most common cancer and the global leading cause of cancer-related deaths among women (Sung et al., 2021). The surgical treatment of BC has evolved from radical mastectomy to breast conservation therapy. Other treatment modalities have also been developed, such as minimally invasive ablation techniques like cryotherapy, radiofrequency, and microwave and laser ablations (Fleming, Holbrook & Newell, 2017; Peek & Douek, 2017; Brem, 2018; Peek et al., 2017). The advantages of BC ablation treatment include its low treatment cost, infrequent general anesthesia requirement, low complication rates and severities, short recovery time and satisfying cosmetic results, in addition to it being an appropriate option for elderly patients who have comorbidities and are unfit for surgery (Peek et al., 2017; Roubidoux, Yang & Stafford, 2014; Yu et al., 2020).

During the past decade, microwave ablation (MWA) has attracted attention due to its potential advantages over other thermal ablation techniques such as its large target area and the high intratumoral temperature produced by active heating (Yu et al., 2011; Zhou et al., 2012a; Zhou et al., 2014). However, for the MWA procedure to be considered technically successful, the tumor and a large enough safety margin of normal breast tissue both must be included within the ablation zone. The primary problem with using MWA is the difficulty of achieving complete tumor inactivation (Roubidoux, Yang & Stafford, 2014; Yu et al., 2020; Zhou et al., 2012a). Studies have shown that MWA can result in complete tumor destruction in most cases, but residual tumor tissues have remained present in some patients and animal models (Zhou et al., 2012a; Zhou et al., 2012b; Song et al., 2016). In hepatocellular carcinoma (HCC), residual tumors following thermal ablation showed accelerated malignant behaviors and aggressive progression (Liu et al., 2015; Zhao et al., 2018). Residual tumors are the major factor lowering the efficacy and survival time of patients receiving thermal ablation (Medhat et al., 2015; Yu & Burke, 2014). Multifaceted factors have been involved in the more malignant phenotypes of residual tumors and their accelerated local progression in HCC after insufficient thermal ablation. For example, Liu et al. (2015) reported that sublethal heat treatment promoted HCC cell proliferation through an increased expression of vascular endothelial growth factor (VEGF) by CaMK II-induced ERK activation. Another study showed that insufficient thermal ablation can facilitate the growth and metastasis of residual hepatic VX2 carcinoma cells owing to the induction of over-expressions of VEGF, proliferating cell nuclear antigen (PCNA) and matrix metalloproteinase-9 (MMP-9) (Ke et al., 2010). Cumulative evidence has revealed that sublethal heat treatment can activate the epithelial-mesenchymal transition (EMT) process and provoke a morphological transformation in the surviving HCC cells (Dong et al., 2013; Yoshida et al., 2013). It has been demonstrated that TGF-*β*2 which belong to the family of transforming growth factors often associated with cancer cell invasiveness and the EMT (Lamouille, Xu & Deryn, 2014). Studies have highlighted the role of autophagy activation in the proliferation and progression of HCC after insufficient thermal ablation (Zhao et al., 2018; Jiang et al., 2019a; Jiang et al., 2019b). Only one study has reported that insufficient MWA promotes the EMT of residual BC cells by activating the *β*-catenin signaling pathway, resulting in enhanced distant metastases by the residual BC cells (Kong et al., 2017). However, the role of autophagy in the altered invasion and metastasis capabilities of residual BC cells after insufficient MWA is unknown. A growing body of evidence indicates that EMT and autophagy are two major biological processes in the occurrence and development of cancer, and there is a complex association between the EMT- and the autophagy-correlated signaling pathways (Chen et al., 2019a; Chen et al., 2019b; Gugnoni et al., 2016). This led us to hypothesize that insufficient MWA could induce autophagy, which would in turn promote EMT-caused invasion and metastases by residual BC cells.

In this study, we show evidence for autophagy’s key role in the modulation of the biological status of residual heat-treated BC cells as seen by: (1) BC cells surviving sublethal heat treatment through autophagy induction; (2) autophagy promoting the EMT of the residual BC cells by up-regulating the TGF-*β*_2_/Smad2 signaling; (3) the reciprocal interactions of autophagy and TGF- *β*_2_ promoted-EMT enhancing the invasion and migration capabilities of the residual BC cells in this process; and, (4) an autophagy inhibitor suppressing proliferation and increasing apoptosis of the residual BC cells.

## Materials and Methods

### Cell culture and heat treatment

The human BC cell lines MCF-7 and MDA-MB-231 (MDA-231) were obtained from the Cell Bank of the Chinese Academy of Sciences (Shanghai, China) and cultured in minimal essential medium (MEM) with 10% fetal calf serum (Gibco, Thermo Fisher Scientific, Dreieich, Germany), 2 mM L-glutamine, non-essential amino acids, 1 mM sodium pyruvate, 100 units/mL of penicillin and 100 µg/mL of streptomycin. The cells were maintained in a humidified atmosphere of 5% CO_2_ at 37 °C.

MCF-7 and MDA-231 cells were grown on six-well plates (5 × 10^4^ cells/well), and after 24 h of incubation, they were exposed to heat treatment. Heat treatment was performed by sealing the tops of culture flasks with parafilm and submerging the flasks in an isothermic water bath set to the target temperatures of 37 °C, 42 °C, 45 °C, 47 °C, 50 °C, 53 °C and 55 °C for 30 min. Next, the cells were seeded into 96-well culture plates (1 ×10^4^ cells per well) in 100 µL DMEM and maintained at 37 °C for 24 h for recovery. The culture medium was exchanged every 8 h for 24 h to remove dead cells and debris. One day after heat treatment, cell viability was measured using a CCK-8 assay according to the manufacturer’s instructions. We used a nonlinear regression curve fitting by Prism 6.0 (GraphPad Software; GraphPad, Inc., San Diego, CA, USA) to calculate IT_50_, the temperature that induced a 50% reduction in cell viability relative to that in the 37 °C control cells, . The IT_50_ data were used for subsequent experiments to simulate sublethal heat treatment conditions. Heat treatment dose–response curves of BC cells showed that IT_50_ was 47.2 °C for MCF-7 and 46.8 °C for MDA-231. Our autophagy detection experiments showed a significant heat-induced autophagy at 47 °C for 30 min. On the basis of these data, we selected 47 °C for 30 min to simulate the effects of insufficient MWA and maintained cells at 37 °C for *in vitro* control experiments. After heat treatment, cells were cultured at 37 °C for recovery.

### Antibodies and reagents

Chloroquine (CQ), 3-methyladenine (3-MA), rapamycin and TGF- *β*_2_ were purchased from Sigma-Aldrich (St Louis, MO, USA). LY2109761 was from Selleck Chemicals (Houston, TX, USA). Antibodies against microtubule-associated protein 1 light chain 3-I (LC3-I), LC3-II, p62, Beclin 1, ATG7, poly (ADP-ribose) polymerase (PARP), cleaved-PARP, caspase 9, cleaved-caspase 9, Bax, and Bcl-x1 were from Abcam (Cambridge, MA, USA). Antibodies against TGF-*β*_2_, Smad2, p-Smad2, E-cadherin, *α*-catenin, *β*-catenin, N-cadherin, fibronectin, ZO-1, Vimentin, MMP-9, Snail, Slug and *β*-actin were from Santa Cruz Biotechnology (Santa Cruz, CA, USA). Primary antibodies of LC3-II and E-cadherin and secondary antibodies Alexa Fluor 568 anti-mouse IgG and Alexa Flour 568 anti-rabbit antibodies were from Jackson Immuno Research (Lancaster, PA, USA).

### Cell viability and clonogenic assays

Cells were seeded into 96-well plates at a density of 1 × 10^4^ cells per well and cultured overnight for attachment. Next, cells were exposed to heat treatment, and/or 3-MA (8 mM) for the indicated times (Dai et al., 2018). After incubation for 24, 48, 72, 96, and 120 h, we determined cell viabilities using a Cell Counting Kit-8 (CCK-8) assay (DoJindo, Tokyo, Japan), according to the manufacturer’s instructions as previously reported (Chen et al., 2019a; Chen et al., 2019b). The absorbance was measured at a wavelength of 450 nm with a reference wavelength of 650 nm using a microplate reader.

We assessed the cell colony formation ability using a clonogenic assay as previously reported (Chen et al., 2019a; Chen et al., 2019b). Briefly, cells were seeded into six-well dishes at a concentration of 1 × 10^3^ cells/well and allowed to grow in complete medium for 14 days after treatment with the indicated temperatures and/or drugs. The colonies obtained were washed with PBS and fixed in 4% paraformaldehyde for 20 min at room temperature, followed by staining with crystal violet. The colonies were counted and the numbers compared with those of untreated cells (control).

### Analysis of cell apoptosis

Cells were treated with the indicated temperature in the absence or presence of 3-MA (10 µM) for 30 min, followed by culture at 37 °C for 24 h. An Annexin V-FITC/ propidium iodide (PI) apoptosis kit (Multi Sciences Biotech, Hangzhou, Zhejiang, China) was used to analyze the apoptosis rate. When the time point had been reached, cells were washed with cold PBS twice. After adding 5 µL Annexin V-FITC and 10 µL PI, cells were analyzed by fluorescence-activated cell sorting (FACS) with flow cytometer, as previously described (Dai et al., 2018). In addition, we detected apoptotic cell features using a Hoechst 33258 staining kit (Beyotime, Shanghai, China). Cells were seeded into a six-well plate (1 × 10^5^ cells/well), cultured for 24 h and, subsequently, treated with the indicated temperature and/or agents. Next, cells were stained with Hoechst 33258. Apoptotic morphological features (chromation condensation and nuclear fragmentation) were evaluated and imaged using fluorescent microscopy.

### Western blotting and real-time quantitative PCR analysis

Before and after treatment with the indicated temperature and/or agents such as CQ (10 µM), 3-MA (8 mM) and LY2109761 (20 µM), or transfections with the siRNA against relevant genes, we prepared whole lysate proteins extracted from the cells and analyzed them using standard western blotting as previously described (Dai et al., 2018).

For real-time quantitative PCR analyses, total RNA was extracted from cell samples using Trizol reagents (Invitrogen, Carlsbad, CA, USA) following the manufacturer’s instructions. RNA (1–2 µg) were reversely transcribed into cDNA using the Super Script III First-Strand Synthesis kit (Invitrogen, Carlsbad, CA, USA) according to the manufacturer’s instructions; and the reactions were run on a BAI 7500 Fast real-time PCR system, and performed with the following conditions: 95 °C for 3 min; 95 °C for 10 s, and 40 amplification cycles (95 °C for 10 s and 65 °C for 30 s, and 60 for 15 s). The reaction system consisted of 20 µL, including 6.4 µL DNase-free (dd) water, 10 µL Mix SYBR (QP-010140 FOREGENE), 0.8 µL forward and reverse primers and 2 µL cDNA diluent. GAPDH was used as the internal control. Detection of PCR products was accomplished by measuring the emitting fluorescence (Rn) at the end of each reaction step. mRNA relative quantification was calculated using the Ct(2^−ΔΔCt^) method (VanGuilder, Vrana & Freeman, 2008). The sequence of primers used in qrt-PCR are shown in Table S1.

### Small interfering RNA transfection

All siRNA reagents used in this study were purchased from Guangzhou Ribobio (Guangzhou, China). Before treatment with the indicated temperatures, cells were transfected with Lipofectamine 2000 reagent according to the manufacturer’s instructions as previously published (Chen et al., 2019a; Chen et al., 2019b). The transfection efficacies were verified using western blotting.

### Immunofluorescence and monodansylcadaverine staining

LC3-II puncta were detected *via* immunofluorescence as previously published (Dai et al., 2018; Xiang et al., 2020). Briefly, cells were seeded into 96-well plates and treated with the indicated temperature or/and agents before being fixed with 2% paraformaldehyde in PBS for 20 min at 37 °C. Following permeabilization for 3 min with PBS−0.2% Triton X-100, the cells were incubated overnight at 4 °C with anti-LC3 antibody diluted 1:100. Next, the cells were incubated for 1 h at room temperature with Alexa Fluor 488-conjugated secondary antibody at a 1:400 dilution. The cells were counterstained with Hoechst 33342 for 5 min for nuclear staining, and mounted onto glass slides. We visualized the cells using a Leica confocal laser scanning microscope (Leica, Wetzlar, Germany).

The formation of acidic vascular organelles (AVOs) was detected using monodansylcadaverine (MDC) staining as previously reported (Dai et al., 2018; Xiang et al., 2020). Briefly, cells were seeded onto 24-well plates and treated with the indicated temperature or/and agents. Next, the cells were incubated with 0.5 mM MDC for 2 h in serum-free medium at room temperature. We detected cellular fluorescence changes at an excitation of 380 nm and an emission of 510 nm as observed under an epi-fluorescence microscope (Olympus, Tokyo, Japan).

For the detection of the intracellular localization of proteins, cells were seeded on gelatin-coated coverslips and treated with the indicated temperature and/or agents. Next cells were fixed with 4% paraformaldehyde for 15 min, permeabilized with 0.1% Triton X-100 for 10 min and blocked with 0.5% BSA for 30 min. Target proteins in cells were visualized after incubation with the corresponding antibodies overnight, followed by incubation with FITC-conjugated secondary antibodies for 1 h. We subsequently used rhodamine-conjugated phalloidin and 4, 6-diamidino-2-phynylindol (DAPI) to localize F-actin and nuclei. Images were acquired with a confocal microscope (Leica, Wetzlar, Germany).

### Transmission electron microscopy (TEM)

Autophagosomes were observed using TEM as previously reported (Dai et al., 2018). Briefly, cells (10 × 10^6^ cells/mL) were treated with the indicated temperatures and fixed with Karanovsky’s fixation for 1 h at room temperature. Dehydration was performed with graded ethanol and propylene oxide, and cells were embedded in araldite using a kit (Merck, Rahway, NJ, USA). Ultrathin sections were prepared on an LKBIII Ultratome using a diamond knife (Diatome, Switzerland), and the sections were mounted on Formvar-coated 200-mesh nicked grids. The grids were double stained for 1 h with saturated uranyl acetate in 50% methanol. The sections were examined in a JEOL-100 CX transmission electron microscope, at 80 kV.

### Cell migration and invasion assays

We assessed cell migration using wound healing assays as previously reported (Xiang et al., 2020). After heat treatment, cells were cultured for 24 h and grown in medium containing 10% FBS to form nearly confluent cell monolayers. We used pipette tips to carefully scratch the surface creating a denuded zone (gap) of constant width. Subsequently, we washed the cellular debris with PBS. The wound closure was monitored and photographed at 0 and 72 h under a Levia inverted microscope (Olympus, Tokyo, Japan). To quantify the migrated cells, pictures of the initial wounded monolayers were compared with the corresponding pictures of cells at the end of the incubation period.

We assessed cell invasion using a Transwell membrane culture system coated with Matrigel as previously reported (Xiang et al., 2020). After treatment with the indicated temperature and/or agents, cells were seeded on the upper wells of a precoated Transwell plate (2 × 10^4^ cells per well). The lower wells of each Transwell plate contained the same medium with 10% FBS. After 48 h of incubation, the cells on the upper well and the membranes coated with Matrigel were swabbed with a Q-tip, fixed with methanol, and stained with 20% Giemsa solution. The cells attached to the lower surface of the polycarbonate filter were counted under an inverted microscope (we determined that the percentage of invading control cells to be 100%).

### *In vivo* xenograft tumor assay

Female BALB/c nude mic (specific pathogen-free grade, 5–6 weeks of age) were obtained from the Animal Core Facility of Nanjing Medical University (Nanjing, China) and maintained under standard pathogen-free conditions. All animal procedures were performed in accordance with the protocols approved by the Institutional Animal Care and Use Committee of the Jiangsu University (JSDX-2022-068). MDA-231 cells were pretreated with 47 °C for 30 min or maintained at 37 °C (control). After recovery at 37 °C for 24 h, cells (5 × 6^6^ cells in 100 µl of serum-free RPMI 1640 medium) were subcutaneously injected into the mammary fat pad of 20 nude mice. Mice were divided into four group (*n* = 5 per group): vehicle (37 °C treatment), CQ (37 °C treatment), heat treatment (47 °C), and a combination of CQ and heat treatment. Starting on the second day, mice in the vehicle and heat treatment groups received intraperitoneal injections of 100 µl of PBS once every other day, while mice in the CQ and combination treatment groups received intraperitoneal injections of 60 mg/kg CQ in 100 µl of PBS once every other day for a total of 14 injections. Tumor size was measured once per week for 6 weeks, and tumor volumes were calculated using the formula: V =(Length × Width^2^)/2. By the end of the experiment, mice were euthanized, the tumors were excised and weighed, and half of the tumor samples were fixed for histological examination. The remaining tumor samples were stored at −80 °C for western blotting.

### Tail vein metastatic assays

MDA-231 cells pretreated with 47 °C and maintained at 37 °C were suspended in 100 µl PBS and injected through the tail vein into female BALB/c nude mice. Mice were divided into three group (*n* = 5 per group): vehicle (37 °C treatment), heat treatment (47 °C) and a combination of heat treatment and CQ. One day after tail vein injection, mice in the vehicle and heat treatment groups received intraperitoneal injections of 100 µl of PBS thrice weekly, while mice in the combination treatment group received intraperitoneal injections of 60 mg/kg CQ in 100 µl of PBS thrice weekly. Five weeks after the injection treatment, the mice were sacrificed and the lung tissues were isolated and made into serial sections for hematoxylin & eosin (HE) staining and observed under a light microscope for counting the number of pulmonary metastatic foci.

### Statistical analysis

We performed all the experiments in triplicate, and the results are presented as means ± SDs. To compare the two groups, we used a two-tailed unpaired *t*-test. To compare multiple groups, we applied one-way ANOVA analyses followed by Tukey post hoc tests. *p*-values <0.05 were considered significant. All statistical data were analyzed using SPSS (version 24.0) or GraphPad Prism (7.0 version) software.

## Results

### Sublethal heat treatment induces apoptosis but promotes proliferation of surviving BC cells

**Figure 1:**
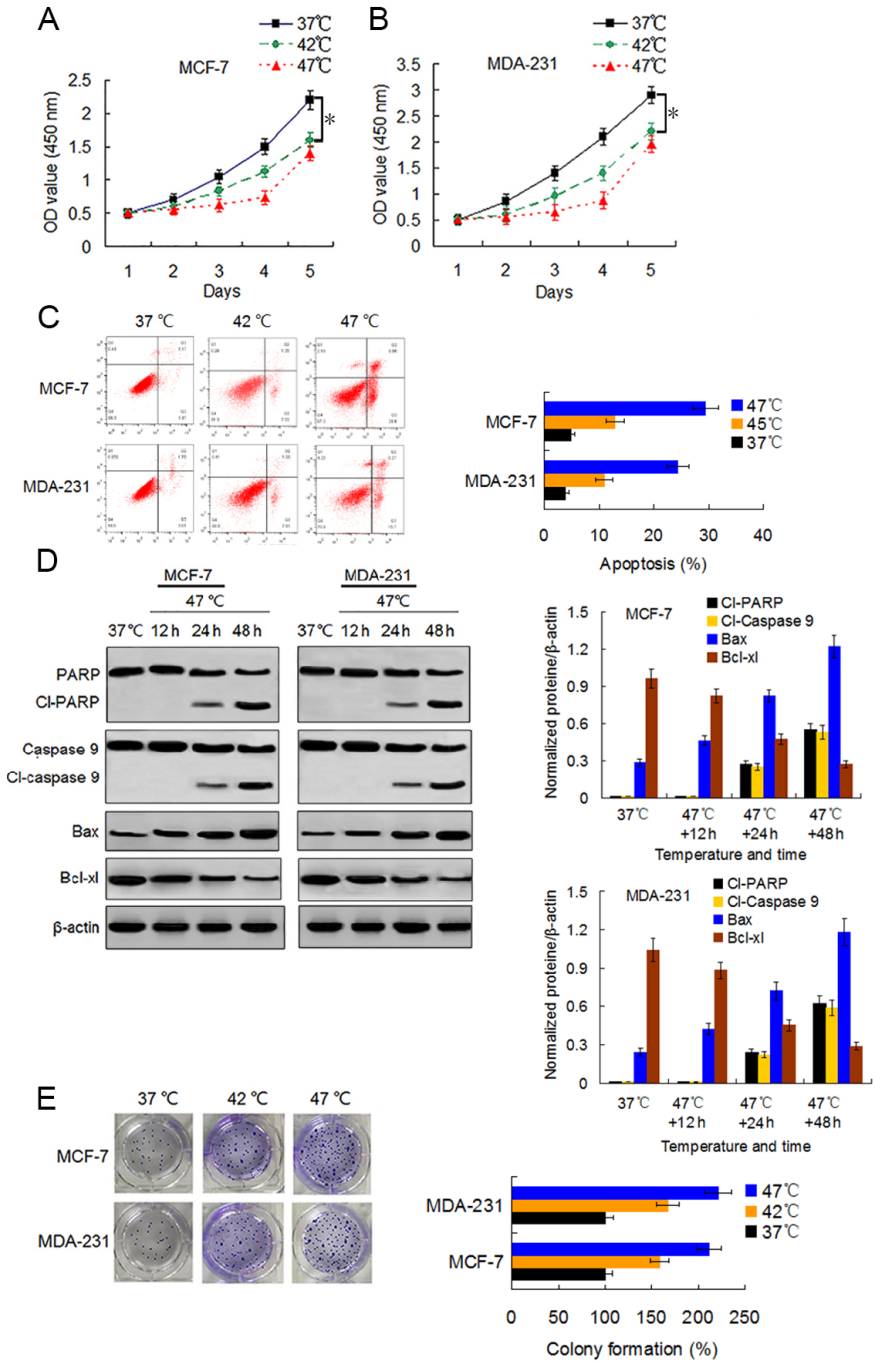
Heat treatment reduces viability and induces apoptosis but promotes proliferation of surviving BC cells. (A and B) MCF-7 and MDA-231 cells were exposed to 42 °C and 47 °C for 30 min, followed by culture at 37 °C for 24 h (or they were maintained at 37 °C for the control experiment) before testing the cell viability at the indicated time points using a CCK-8 assay. *P < 0.05. (C) MCF-7 and MDA-231 cells were treated as (A and B), and the apoptotic rates were analyzed 48 h later *via* flow cytometry. *P < 0.05 *vs* 42 °C, *P < 0.005 *vs* 37 °C. (D) The two cell lines were exposed to 47 °C for 30 min, followed by culture at 37 °C for 48 h (or they were maintained at 37 °C for the control experiment), and the expressions of the indicated proteins were detected at the indicated time points using western blots. (E) MCF-7 and MDA-231 cells were exposed to 42 °C and 47 °C for 30 min, followed by culture at 37 °C for 24 h (or they were maintained at 37 °C for the control experiment); next, cultures were maintained for 14 days. The colony formation abilities were assessed *via* clonogenic assays. *P < 0.01 *vs* 42 °C, *P < 0.001 *vs* 37 °C.

We first examined the viability changes of BC cells at different time points following heat treatment at different temperatures to evaluate the influence of heat treatment on the proliferation of BC cells. MCF-7 and MDA-231 cells were exposed to heat treatment of 42 °C and 47 °C, respectively, for 30 min to simulate insufficient MWA conditions. We used cells incubated at 37 °C for the control experiments. Thereafter, heat-treated cells were cultured at 37 °C for recovery. After continuous incubation at 24, 48, 72, 96, and 120 h, we determined the cells viabilities using a CCK-8 assay at the corresponding time points. The viability rates of the cells treated at 42 °C and 47 °C decreased gradually in a temperature-dependent manner during the first four days as compared to the viability rates of the control cells. However, cells treated at 47 °C showed an increase in their viability rate on the fifth day after the heat treatment (Figs. 1A and 1B). Moreover, heat treatment induced a temperature-dependent cell apoptosis (Fig. 1C), which was paralleled by an increase in cleaved-PARP, cleaved-caspase 9, and Bax, and by a decrease in Bcl-xl in a time-dependent manner (Fig. 1D). It is worth nothing that heat-treated cells displayed more colony formations than cells treated at 37 °C (Fig. 1E). The data suggest that heat treatment promoted a delayed proliferation of the BC cells, although the cell viability rates were reduced shortly after the heat treatment.

### Sublethal heat treatment induces autophagy of BC cells

**Figure 2:**
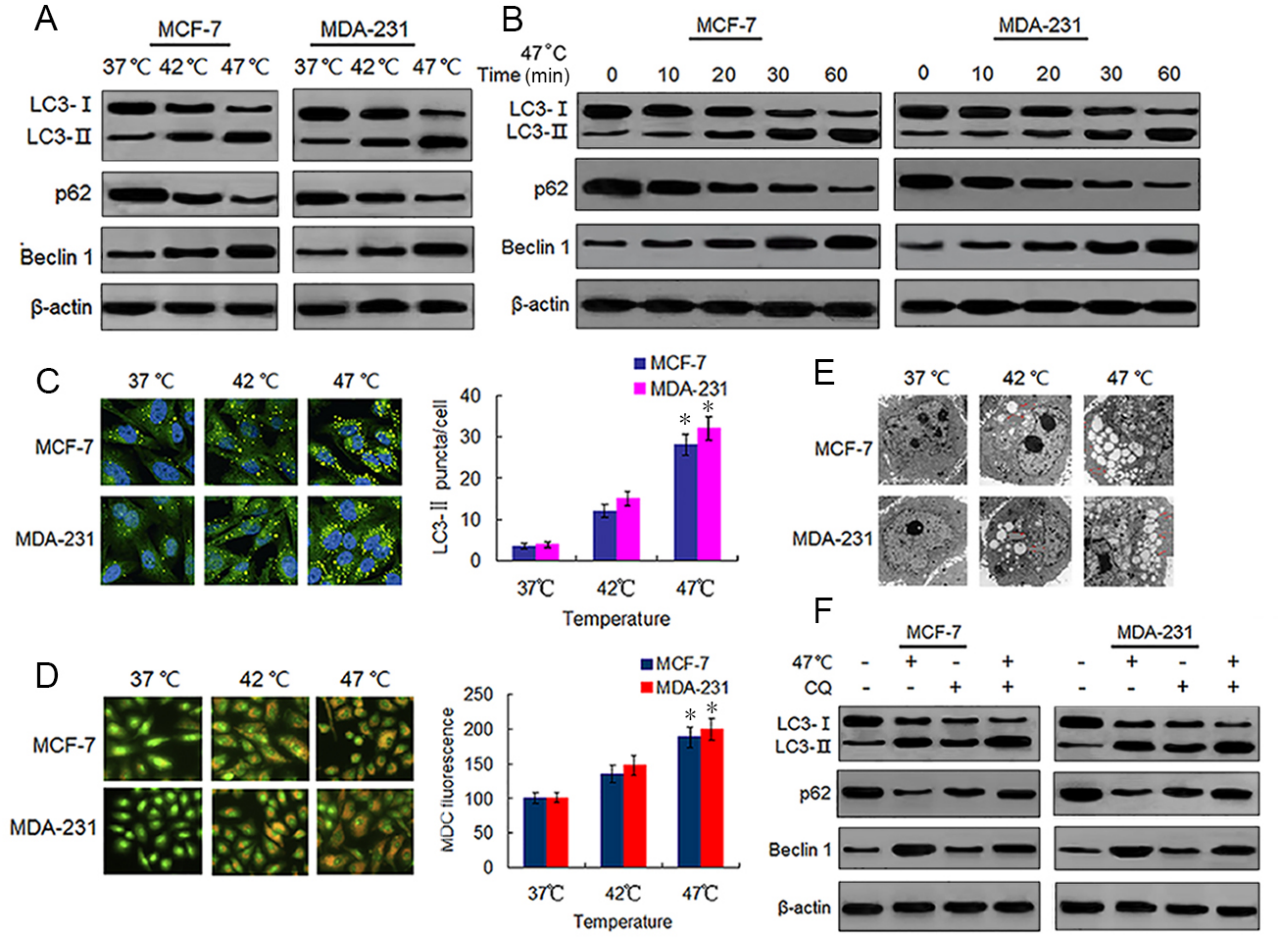
Heat treatment induces autophagy in BC cells. (A) MCF-7 and MDA-231 cells were exposed to 42 °C and 47 °C for 30 min, followed by incubation in 37 °C for 24 h (or they were maintained at 37 °C for the control experiment), then the expressions of the indicated proteins were analyzed using western blots. (B) MCF-7 and MDA-231 cells were treated with 47 °C for 30 min, and the expressions of the indicated proteins were analyzed using western blots at the indicated time points. (C) The two cell lines were treated as (A, B), then LC3-II puncta formation was detected using immunofluorescence analysis and imaged with a confocal microscope (left). The number of LC3-II puncta/cells were quantified using Image-Pro plus 5.1 software (right). *P < 0.05 *vs* 42 °C, *P < 0.005 *vs* 37 °C. (D) The two cell lines were treated as (A, B), then AVOs in cells were detected by MDC staining and imaged with a fluorescence microscope (left). We defined the intensity of MDC fluorescence in control cells (37 °C) as 100%. *P < 0.05 *vs* 42 °C, *P < 0.001 *vs* 37 °C. (E) Transmission electron microscopy showed an increased number of autophagic vacuoles in MCF-7 and MDA-231 cells after exposure to 47 °C for 30 min. (F) MCF-7 and MDA-231 cells were exposed to 47 °C for 30min in the absence or presence of CQ (10 µM), followed by culture at 37 °C for 24 h, the expressions of the indicated proteins were analyzed using western blots.

Sublethal heat treatment is known to induce autophagy in Hela, A549 and HCC cells (Jiang et al., 2019a; Jiang et al., 2019b; Zhao et al., 2009). We tested whether this phenomenon also occurred in BC cells by detecting autophagy-associated protein changes in surviving heat-treated BC cells. We observed increased protein expressions of LC3-II and Beclin 1, and decreased protein expression of p62 in heat-treated BC cells as compared with the same expressions in control cells (Fig. 2A). We confirmed these protein expression changes in a time-dependent manner using western blotting (Fig. 2B). We next analyzed autophagosome formation *via* LC3-II immunofluorescence staining. Heat treatment increased the formation of autophagosomes in a temperature-dependent manner as indicated by an increase in the number of LC3-II-positive puncta in MCF-7 and MDA-231 cells (Fig. 2C). The MDC staining of heat-treated cells showed an increase in temperature-dependent fluorescence intensity (Fig. 2D), indicative of the presence of acidic vesicular organelles (AVOs) including lysosomes and autolysosomes. Further examination using TEM revealed an increased number of autophagosomes in heat-treated BC cells (Fig. 2E). An increase of LC3-II protein levels can result from an upregulation of autophagosome formation (stimulated flux) as well as a blockage of autophagosome degradation (interrupted flux). An increase in LC3-II protein levels between conditions when flux is briefly blocked with an inhibitor of autophagosome degradation is indicative of upregulated autophagy (Klionsky et al., 2016). Hence, we treated cells with a autophagy inhibitor, CQ, which inhibits late stage autophagy through the suppression of autophagosome degradation by inhibiting lysosomal acidification (Pasquier, 2016). Our results showed that CQ treatment resulted in the further accumulation of LC3-II in the heat-treated cells (Fig. 2F), indicating that sublethal heat treatment induced the increase of autophagosome formation rate and autophagy flux in BC cells.

### Heat-induced autophagy promotes EMT in BC cells by up-regulating TGF-*β*2/Smad2 signaling

**Figure 3:**
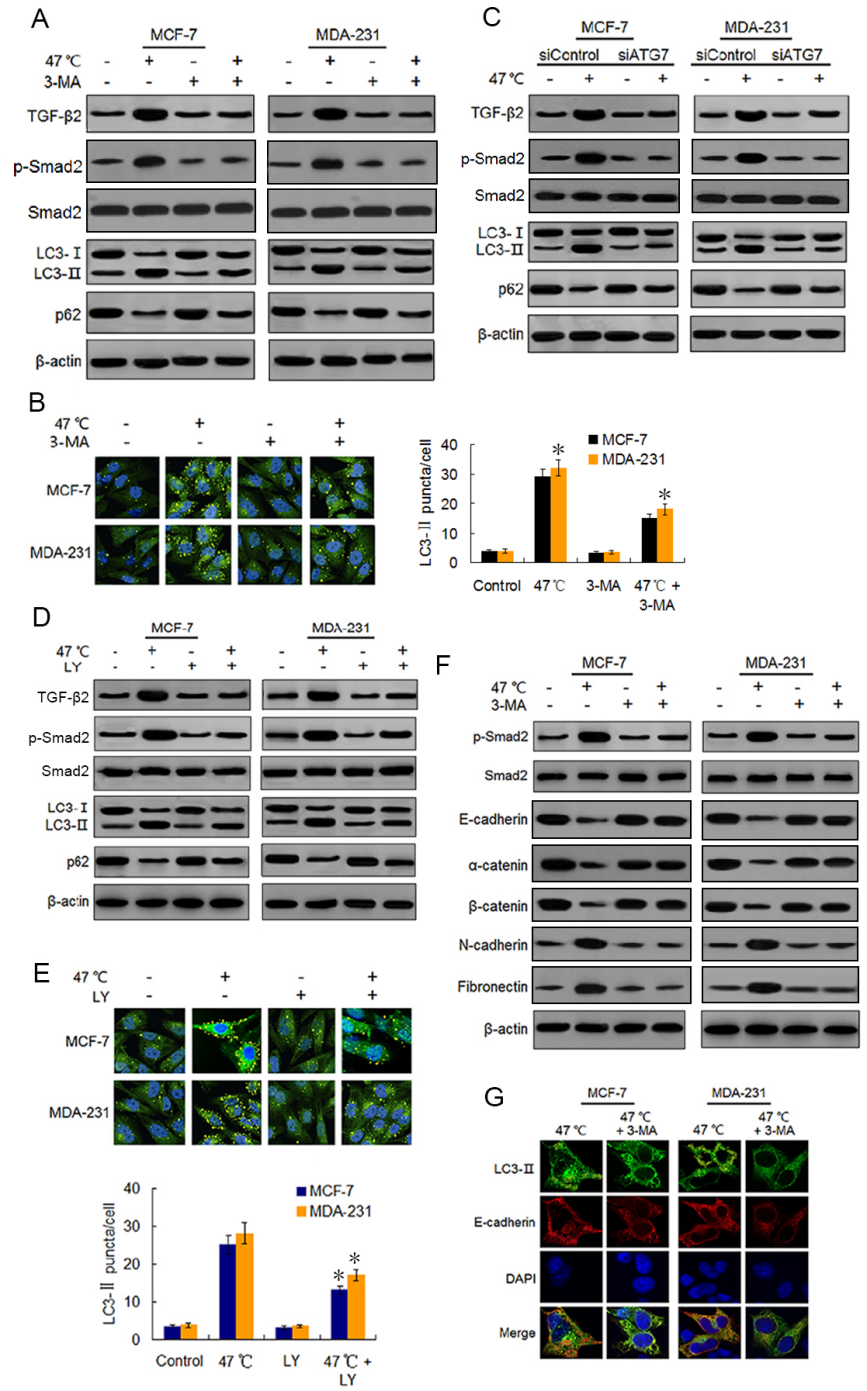
Heat-induced autophagy promotes EMT in BC cells by up-regulating TGF- *β*2. (A) MCF-7 and MDA-231 cells were exposed to 47 °C for 30 min in the absence or presence of 3-MA (10 µM), followed by culture at 37 °C for 24 h (or they were maintained at 37 °C for the control experiment). Then expressions of the indicated proteins were analyzed using western blots. (B) MCF-7 and MDA-231 cells were treated as (A), the LC3-II puncta formation was determined *via* immunofluorescence analysis and imaged with a confocal microscope (left). The number of LC3-II puncta/cell was quantified using Image-Pro plus 5.1 software (right). *P < 0.01 *vs* 47 °C. (C) After transfection with siATG7 or siControl, MCF-7 and MDA-231 cells were exposed to 47 °C, followed by culture at 37 °C for 24 h (or they were maintained at 37 °C for the control experiment). The expressions of the indicated proteins were analyzed using western blots. (D) The two cell lines were exposed to 47 °C for 30 min in the absence or presence of LY2109761 (20 µM), or they were maintained at 37 °C for the control experiment, the expressions of the indicated proteins were analyzed using western blotting. (E) The two cell lines were treated as (D), the LC3-II puncta formation was determined *via* immunofluorescence analysis and imaged using a confocal microscope (upper). The number of LC3-II puncta/cell was quantified using Image-Pro plus 5.1 software (lower panel). *P < 0.05 *vs* 47 °C. (F) The two cell lines were treated as (A), and then fixed with methanol; immunostained with anti-LC3-II (green), anti-E-cadherin (red), and DAPI (blue) antibodies; and observed under a confocal microscope to show the intracellular co-localization of LC3-II and E-cadherin. (G) MCF-7 and MDA-231 cells were treated as (A), and the expressions of the indicated proteins were analyzed using western blots.

Autophagy has been reported to be necessary for TGF-*β*2-induced EMT through Smad2 signaling in HCC cells (Dash et al., 2018). Therefore, we conjectured whether the heat-induced autophagy would facilitate EMT in BC cells by up-regulating the TGF-*β*2/Smad2 signaling pathway. We examined the effects of autophagy induction or inhibition on TGF-*β*2 and EMT, and we explored the reciprocal role between autophagy and TGF-*β*2 during the process. We found that heat treatment activated autophagy and also up-regulated the TGF-*β*2 expression and phosphorylation level of Smad2. Co-treatment with 3-MA, an early phase autophagy inhibitor that can impedes autophagosome formation *via* its inhibitory effect on class III PI3K (Pasquier, 2016; Miller et al., 2010), suppressed both heat-induced autophagy, TGF-*β*2 expression and Smad2 phosphorylation (Figs. 3A and 3B). We obtained similar results with the BC cells co-treated with ATG7 knock-down and heat exposure (Fig. 3C; Figs. S1A) and S1B). To inhibit the TGF-*β*2 signaling pathway in BC cells, we co-treated the cells with heat exposure and LY2109761, a selective TGF-*β*2 receptor type I/II inhibitor (Yang et al., 2009). Our results showed that LY2109761 attenuated the heat treatment-induced TGF-*β*2 upregulation and inhibited Smad2 phosphorylation, while also suppressing autophagy induction (Figs. 3D and 3E). Moreover, we treated the BC cells with TGF-*β*2 to directly stimulate the FGF- *β*2/Smad2 pathway, and we observed a significant increase in Smad2 phosphorylation and autophagy levels, which was similar to the results in the cells treated with rapamycin (20 nM), an autophagy inducer (Figs. S2).

Because TGF-*β*2 is a well-known EMT inducer (Kim et al., 2015), we next detected EMT molecule marker expressions following heat treatment using western blots and real-time PCR. Our findings revealed that heat treatment clearly increased the protein expressions of mesenchymal and promigratory markers, such as N-cadherin, fibronectin, vimentin, snail, slug and MMP-9, and concurrently reduced the protein expressions of epithelial markers such as E-cadherin, *α*-catenin, *β*-catenin and ZO-1 (Fig. 3F; Fig. S3A). Real-time PCR analysis showed similar changes in the transcript levels of these EMT-associated markers after heat treatment (Figs. S3B-S3E). Moreover, co-treatment with 3-MA inhibited the heat treatment-induced EMT-associated marker changes in protein and mRNA levels (Fig. 3F; Figs. S3A–S3E).

Using immunofluorescence, we found LC3-II and E-cadherin colocalized in the perinuclear regions after heat treatment unless 3-MA was added (Fig. 3G), suggesting that heat-induced autophagy may entrap E-cadherin from the plasma membrane into autophagosomes for degradation by lysosomes. Taken together, our findings indicate that heat treatment-induced autophagy up-regulates TGF-*β*2/Smad2 signaling and thereby promotes the EMT of BC cells, while at the same time the upregulated TGF-*β*2 facilitates autophagy induction.

### Heat-induced autophagy enhances the migration and invasion capabilities of BC cells through TGF-*β*2-mediated EMT

**Figure 4:**
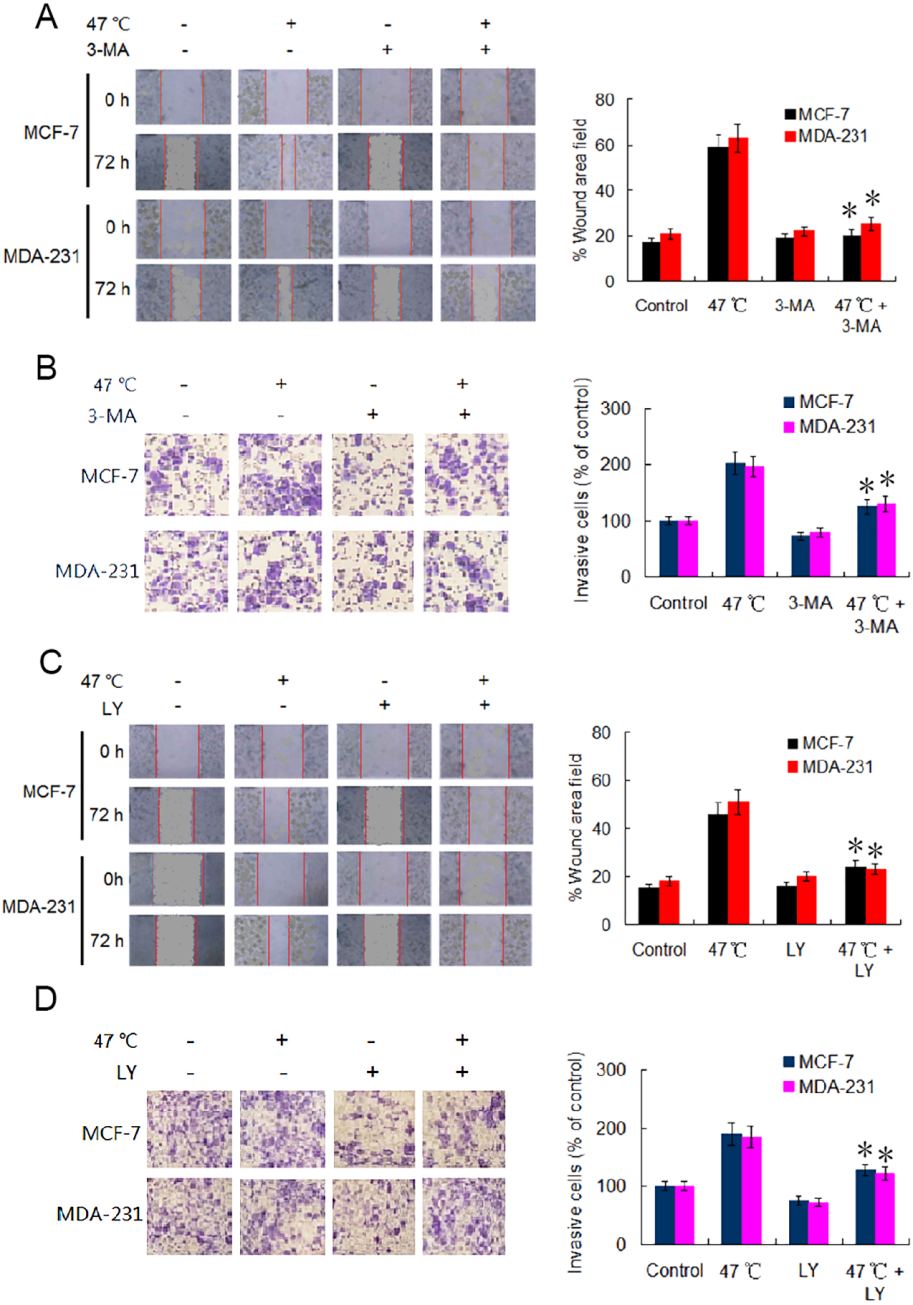
Heat treatment enhances migration and invasion capabilities *via* TGF- *β*2 mediated EMT. (A) MCF-7 and MDA-231 were exposed to 47 °C for 30 min in the absence or presence of 3-MA (10 µM), followed by culture at 37 °C for 24 h (or the cells were maintained at 37 °C for the control experiment); the cell migration ability was measured using a wound-healing assay. Images were acquired immediately (0 h) and at 72 h after wounding. Wound closure was analyzed across five random fields using ImageJ Software to calculate the percentage of wound area filled. *P < 0.01 *vs* 47 °C. (B) MCF-7 and MDA-231 cells were treated as (A), cell invasion ability was measured using a Transwell invasion assay. The invading cells were counted in five randomly-selected areas under a high magnification microscope (200X). We quantified the invasion of control cells as 100% (37 °C). *P < 0.05 *vs* 47 °C. (C) The two cell lines were exposed to 47 °C for 30 min in the absence or presence of LY2109761 (20 µM), followed by culture at 37 °C for 24 h (or they were maintained at 37 °C for the control experiment), and then cell migration ability was measured using a wound-healing assay. *P < 0.01 *vs* 47 °C. (D) The two cell lines were treated as (C), cell invasion ability was measured using a Transwell invasion assay. *P < 0.05 *vs* 47 °C.

EMT is considered a major driver of cancer exacerbations from initiation to metastasis and invasion (Saitoh, 2018). To further verify the occurrence of EMT, we evaluated the migration and invasion of BC cells using wound healing and Transwell assays. Our results indicate that the cell migration and invasion capabilities were markedly enhanced in heat-treated BC cells compared with those in control BC cells (Figs. 4A–4D). Adding 3-MA significantly depressed the cell migration and invasion caused by the heat treatment (Figs. 4A and 4B). Likewise, co-treatment with LY2109761 dramatically restrained the migration and invasion caused by the heat treatment in BC cells (Fig. 4C and 4D). Collectively, these results signify that autophagy induction and TGF-*β*2 upregulation, as well as the interaction between the two signaling pathway, promoted EMT and enhanced the migration and invasion capabilities of heat-treated BC cells.

### Suppressing heat-induced autophagy facilitates apoptosis and decreases proliferation of BC cells

**Figure 5:**
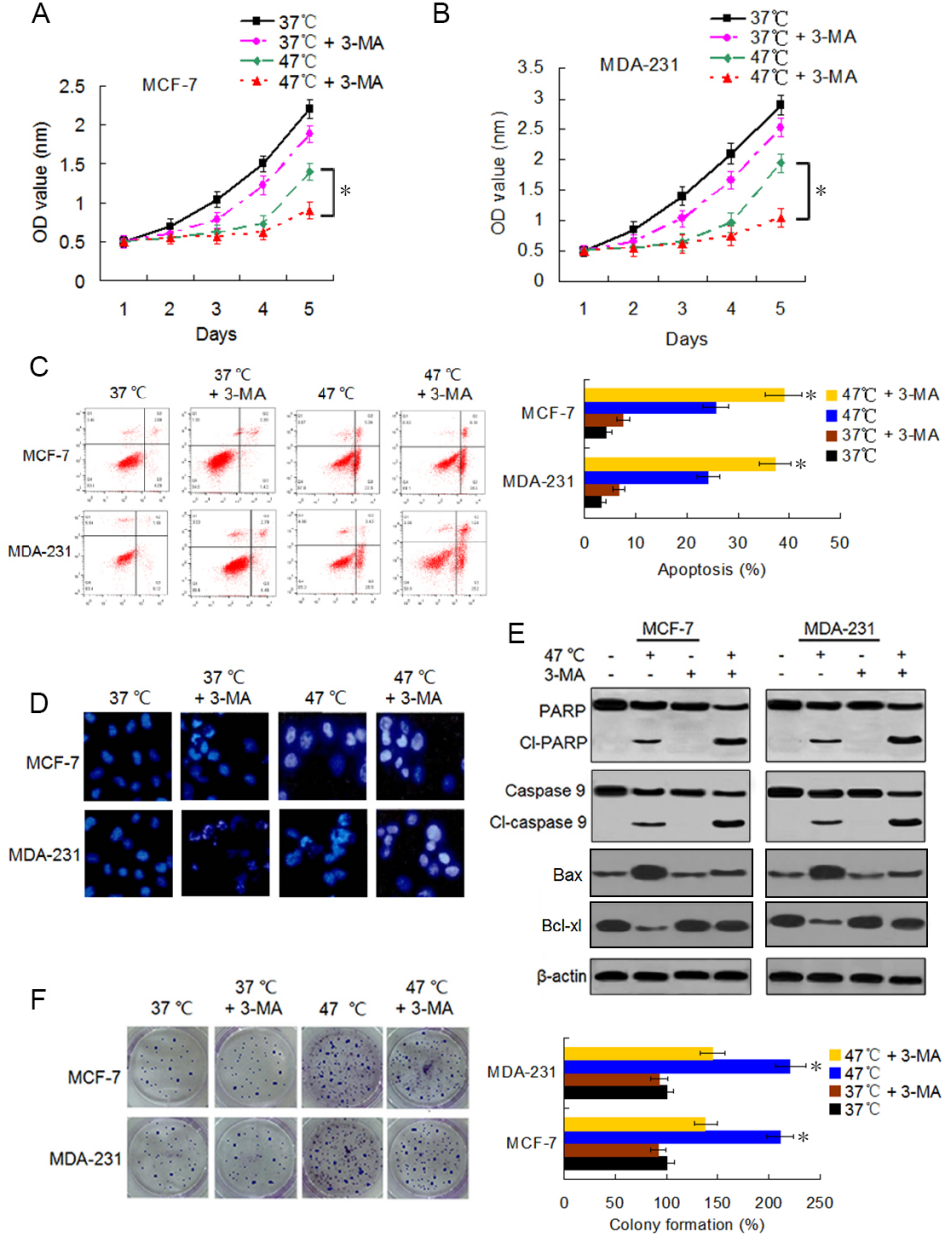
Suppressing heat-induced autophagy facilitates apoptosis and inhibits proliferation in BC cells. (A and B) MCF-7 and MDA-231 cells were exposed to 47° C for 30 min in the absence or presence of 3-MA (10 µM), or they were maintained at 37 °C for the control experiment, followed by culture at 37 °C for 24 h. The viability rate of the cells was detected at the indicated time points using a CCK-8 assay. *P < 0.05. (C) MCF-7 and MDA-231 cells were treated as in A and B, and the apoptotic rate was analyzed at 24 h after treatment using flow cytometry. *P < 0.05 *vs* 47 °C; *P* < 0.001 *vs* 37 °C + 3-MA. (D) The two cell lines were exposed to 37 °C or 47 °C for 30 min in the absence or presence of 3-MA (10 µM), then stained by Hoechst 33258 and imaged using fluorescence microscopy. (E) The two cell lines were treated as in D, the expressions of the indicated proteins were analyzed using western blots. (F) The two cell lines were exposed to 37 °C or 47 °C for 30 min in the absence or presence of 3-MA (10 µM), followed by culture at 37 °C for 14 days, colony formation abilities were assessed using a clonogenic assay. *P < 0.01 *vs* 47 °C + 3-MA.

Accumulating evidence indicates that autophagy serves a cytoprotective role particularly during cancer treatment. In addition, autophagy inhibition can enhance therapy-induced cell death or apoptosis (Amaravadi et al., 2007; Ryter, Cloonan & Choi, 2013). We used the autophagy inhibitor 3-MA to suppress autophagosome formation and block heat-induced autophagy to determine the biological role of autophagy in heat-induced apoptosis. BC cells were treated with 3-MA, heat exposure, a combination of both. As shown in Figs. 5A–5C, co-treatment with 3-MA further reduced the cell viability rates 5 days after heat treatment and further raised the heat treatment-induced cell apoptotic rates. Cells co-treated with 3-MA exhibited more significant apoptotic morphological features such as chromatin condensation and nuclear fragmentation under enhanced bright blue fluorescence (Fig. 5D). In concurrence with these observations, the heat treatment-induced expressions of cleaved-PARP and -caspase 9 were further augmented in the presence of 3-MA (Fig. 5E). The heat treatment-increased Bax expression and -reduced Bcl-xl expression were intensified after 3-MA addition (Fig. 5E). In addition, co-treatment with 3-MA decreased the heat treatment-induced potentiation of cell proliferation (Fig. 5F). All these results demonstrate that autophagy inhibits heat treatment-induced apoptosis, thereby promoting cell proliferation.

### Autophagy inhibitor potentiates the antitumor effect of heat treatment and suppresses heat treatment-promoted metastasis in BC cells

**Figure 6:**
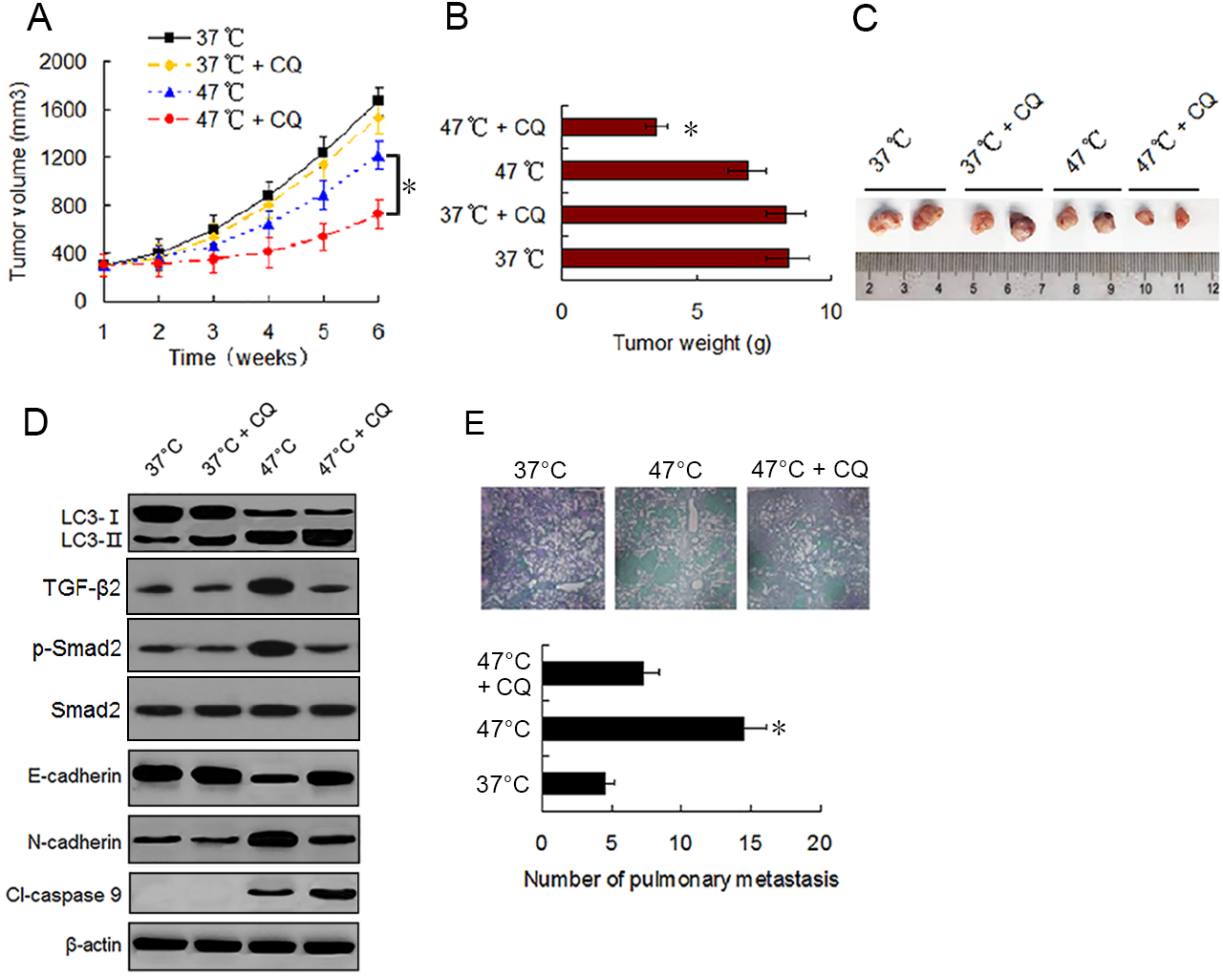
Autophagy inhibitor potentiates the antitumor effect of heat treatment and depresses heat treatment-induced metastasis of BC cells *in vivo*. (A and B) MDA-231 cells pretreated with 47 °C for 30 min or maintained at 37 °C were subcutaneously injected into the mammary fat pad of female BALB/c nude mice. The mice receiving different pretreated cells were treated with or without CQ, respectively. Tumor volume was measured at the indicated time points after the onset of treatment (A) (*P < 0.05). The tumor weight was measured at the end of the experiment (B) (*P < 0.05 *vs* 47 °C). (C) Representative picture of tumor samples from the mice receiving different treatment as indicated. (D) Expressions of proteins as the indicated determined by western blotting in MDA-231 cell tumor samples from the mice receiving different treatment. (E) MDA-231 cells pretreated with 47 °C for 30 min or maintained 37 °C were injected through tail vein into female BALB/c nude mice. The mice received cells pretreated with 47 °C were treated with or without CQ. After four weeks, the mice were sacrificed and the lung tissues were isolated. The lung tissues were sectioned serially and stained with HE. Representative images of HE staining of pulmonary metastatic foci (upper). The number of metastatic foci were counted (lower) (*P < 0.05 *vs* 47 °C).

To evaluate the effect of sublethal heat treatment on tumor growth and determine whether autophagy inhibitor can enhance the antitumor effect of heat treatment *in vivo*, we established a xenograft tumor model of MDA-231 cells and examined the effect of vehicle, CQ, heat treatment alone, and a combination of heat treatment and CQ on tumor growth in nude mice. By the end of *in vivo* experiment, no significant difference in tumor volumes and weights was found between mice treated with vehicle and mice treated with CQ alone. Tumor growth was moderately inhibited by heat treatment alone. However, significant growth inhibition was observed under the combination treatment (Figs. 6A–6C). Western blotting of tumor samples showed that the combination treatment with CQ increased heat treatment induced the accumulation of LC3-II and cleaved-caspase 9, and retarded heat treatment induced the up-regulation of TGF-*β*2, Smad2 phosphorylation and N-cadherin and down-regulation of E-cadherin (Fig. 6D). These data were consistent with the *in vitro* results and further confirmed that autophagy inhibitors can enhance the antitumor effect of heat treatment.

To determine the effect of heat treatment and CQ on the *in vivo* metastasis of MDA-231 cells, a tail vein metastasis assay was applied. As shown in Fig. 6E, the number of metastatic foci in the lung significantly increased in mice receiving heat treated-MDA-231 cells compared with those receiving 37 °C treated-MDA-231 cells, and co-treatment with CQ markedly decreased the number of metastatic foci in lung. The results indicate that sublethal heat treatment promoted the distant metastasis of BC cells, which can be inhibited by suppressing autophagy.

## Discussion

Aggressive local recurrences and accelerated distant metastases by residual tumor cells following insufficient thermal ablation are a major impediment to the application of this technique for the treatment of solid tumors. Therefore, the molecular mechanisms underlying these biological behaviors need to be investigated in depth. In this study, we treated BC cells with sublethal heat exposure to simulate insufficient MWA conditions. Our results show a reduced viability of BC cells and induced apoptosis shortly after heat treatment, whereas the surviving cells exhibited a delayed proliferation raise after the heat treatment. We demonstrated that sublethal heat treatment induced autophagy in BC cells in a temperature- and time-dependent manner, a finding previously reported in HCC cells (Zhao et al., 2018; Jiang et al., 2019a; Jiang et al., 2019b). Importantly, autophagy inhibition by 3-MA suppressed the heat treatment-induced increases in TGF-*β*2 expression, Smad2 phosphorylation and EMTs; thereby repressing the heat-induced migration and invasion by BC cells. These data indicate that autophagy plays a central role in the migration and invasion of BC cells caused by the TGF-*β*2/Smad2-mediated EMT under sublethal heat treatment conditions.

Autophagy is an evolutionarily conserved lysosome-mediated degradation pathway by which cells self-digest selected cellular macromolecules and maintain homeostasis (Yang et al., 2009; Ryter, Cloonan & Choi, 2013). EMT, a switching process from the epithelial phenotype of an adherent cell to a motile mesenchymal phenotype, is known to facilitate cancer metastasis and invasion in several human cancers (Foroni et al., 2012). The EMT signaling pathway can trigger or inhibit autophagy. Furthermore, autophagy is involved in the induction and inhibition of EMT. On the one hand, EMT requires autophagy to support the viability of potential cancer cells metastases (Singla & Bhattacharyya, 2017). On the other hand, increasing additional evidence indicates that autophagy prevents and may suppress EMT in cancer cells (Catalano et al., 2015). Therefore, autophagy plays dual effects on EMT during the development and progression of cancers, depending on the cancer type, stimulus, or cellular context. Thus, there is an interaction between autophagy and EMT (Chen et al., 2019a; Chen et al., 2019b). In this study, we showed that heat treatment-induced autophagy triggered EMT by activating TGF-*β*2/Smad signaling, and we determined the effects of heat treatment-induced autophagy on the migration and invasion of BC cells. We also further explored the relevant underlying mechanisms.

Autophagy promoted the migration and invasion of BC cells by triggering EMT. By applying pharmacological autophagy and TGF- *β*2 receptor inhibitors, we demonstrated that autophagy up-regulated TGF-*β*2 expression and the TGF-*β*2/Smad2 signaling pathway has a role in mediating autophagy-induced EMT. In addition, we revealed that autophagy-induced TGF-*β*2 can promote autophagy. Autophagy and the TGF-*β*2 levels formed a positive feedback loop to synergistically facilitate the migration and invasion of BC cells. These findings also demonstrate that the migration and invasion of BC cells, promoted by autophagy after heat treatment, are dependent on TGF-*β*2/Smad2 signaling. TGF-*β* is considered a master regulator of EMT in carcinoma (Wendt, Allington & Schiemann, 2009). Cancer cells exposed to TGF-*β* can strongly activate autophagy and this effect is primarily facilitated by Smad signaling. However, it was indicated that TGF- *β* can trigger both pro-tumorigenic and anti-tumorigenic signals and the process type may be completely dependent on the cellular context and the stage of tumor progression (Derynck, Akhursk & Balmain, 2001). Nevertheless, at least in the present model, autophagy-upregulated TGF-*β*2 facilitated the migration and invasion of BC cells by mediating EMT after heat treatment.

In this study, another important finding was that of the inhibition of autophagy by 3-MA promoting apoptosis and repressing proliferation in BC cells under heat treatment. These results indicate that heat treatment-induced autophagy plays a protective role against apoptotic cell death. Interactions among various components of the autophagy and apoptosis pathways resulted in a complex crosstalk between the two that was often induced by similar stimuli (Maiuri et al., 2009). For instance, ATG5, which is required for the formation of autophagosomes, also enhances susceptibility to apoptotic stimuli upon cleavage by calpain. The calpain-mediated cleavage of ATG5 switches autophagy into apoptosis, and cleavage of ATG5 results in the translocation of the truncated product from the cytosol to mitochondria, where it triggers cytochrome c release and activation of caspase-dependent apoptosis (Yousefi et al., 2006). In the present model, the precise mechanism underlying the facilitation of apoptosis by autophagy inhibition needs to be further investigated.

Additionally, our *in vivo* mouse model experiments also confirmed the results of the *in vitro* cell model. We found that the heat treatment induced autophagy and activated EMT in MDA-231 cell xenograft tumor, and autophagy inhibition by CQ markedly restrained tumor growth *via* depressing EMT and promoting apoptosis. The tail vein metastasis assay also showed that BC cells exhibited potentiated pulmonary metastasis ability, which could be notably depressed by CQ treatment. Moreover, these data indicate that a single heat treatment can result in a permanent change in BC cell phenotype *in vivo*, as well as producing a stable biochemical change in molecular levels. Other studies have also reported that HCC cells were subjected to sublethal heat treatment for 10 or 30 min and injected into nude mice, and the *in vivo* formed xenograft tumor displayed a continuous change in cell phenotype and changes in expression levels of related genes and proteins (Zhang et al., 2017a; Zhang et al., 2017b; Jiang et al., 2019a; Jiang et al., 2019b).

In conclusion, our data suggest that sublethal heat treatment induces autophagy, which triggers TGF-*β*/Smad2-mediated EMT, thereby promoting proliferation and enhancing the migration and invasion capabilities of the residual BC cells. Aligned with these findings, the inhibition of autophagy enhanced the heat treatment’ anti-BC effects and repressed the potentiation of the BC’ cells’ proliferation and metastatic abilities. Therefore, suppressing autophagy may be an effective strategy for overcoming insufficient MWA ablation-induced progression and metastasis by residual BC cells.

##  Supplemental Information

10.7717/peerj.14640/supp-1 Supplemental Information 1 Knock-down of ATG7 block heat-induced increase LC3-II puncta formation (A) ATG7 expressions in MDA-231 cells were analyzed by western blotting after transfection with siControl, siATG7-1^#^, siATG7-^#^ and siATG7-3^#^. (B) After transfection with siATG7 or siControl, MCF-7 and MDA-231 cells were exposed to 47 °C for 30 min, or maintained at 37 °C, followed by culture at 37 °C for 24 h, then the LC3-II puncta formation was detected using immunofluorescent analysis and imaged by confocal microscope (left). The number of LC3-II puncta/cell was quantified by Image-Pro plus 5.1 software (right) (^∗^*P* < 0.01 *vs* 47 °C + siControl).

10.7717/peerj.14640/supp-2 Supplemental Information 2 TGF- *β*2 induced Smad2 phosphorylation and autophagy MCF-7 and MDA-231 cells were exposed to 47 °C for 30 min, followed by culture at 37 °C for 24 h, or treated with a TGF- *β*2 dose gradient ranging from 1 ng/ml to 10 ng/ml for 24 h, or treated with rapamycin (20 nM) for 24 h, The expressions of the indicated proteins were detected by western blotting.

10.7717/peerj.14640/supp-3 Supplemental Information 3 3-MA attenuate heat treatment-induced changes of EMT marker expressions (A) MCF-7 and MDA-231 cells were exposed to 47 °C for 30 min in the absence or presence of 3-MA (10 µM), followed culture at 37 °C for 24 h. The expressions of the indicated proteins were detected by western blotting. (B–E) MCF-7 and MDA-231 cells were treated as (A), relative levels of the indicated miRNAs were analyzed by real-time quantitative PCR.

10.7717/peerj.14640/supp-4 Supplemental Information 4 Sequences of primers used in quantitative real-time PCR (qrt-PCR) analysis

10.7717/peerj.14640/supp-5 Supplemental Information 5 Raw data of Figs. 1–6 and suppl Figures Raw data include cell viability and clonogenic assay, cell apoptosis assay, rtq-PCR, immunofluorescence staining,and cell migration and invasion assay.

10.7717/peerj.14640/supp-6 Supplemental Information 6 Raw data of WB Raw data of Western blotting include Fig. 1–Fig. 3, Figs. 5 and 6.

## Additional Information and Declarations

### Data Availability

The following information was supplied regarding data availability: The raw data is available in the Supplemental Files.
