# Supplementary figures and images for: Heat treatment-induced autophagy promotes breast cancer cell invasion and metastasis via TGF-β2-mediated epithelial-mesenchymal transitions
Source: PeerJ. 2023 Jan 12;11:e14640. doi: 10.7717/peerj.14640 (PMC9840853; doi:10.7717/peerj.14640)

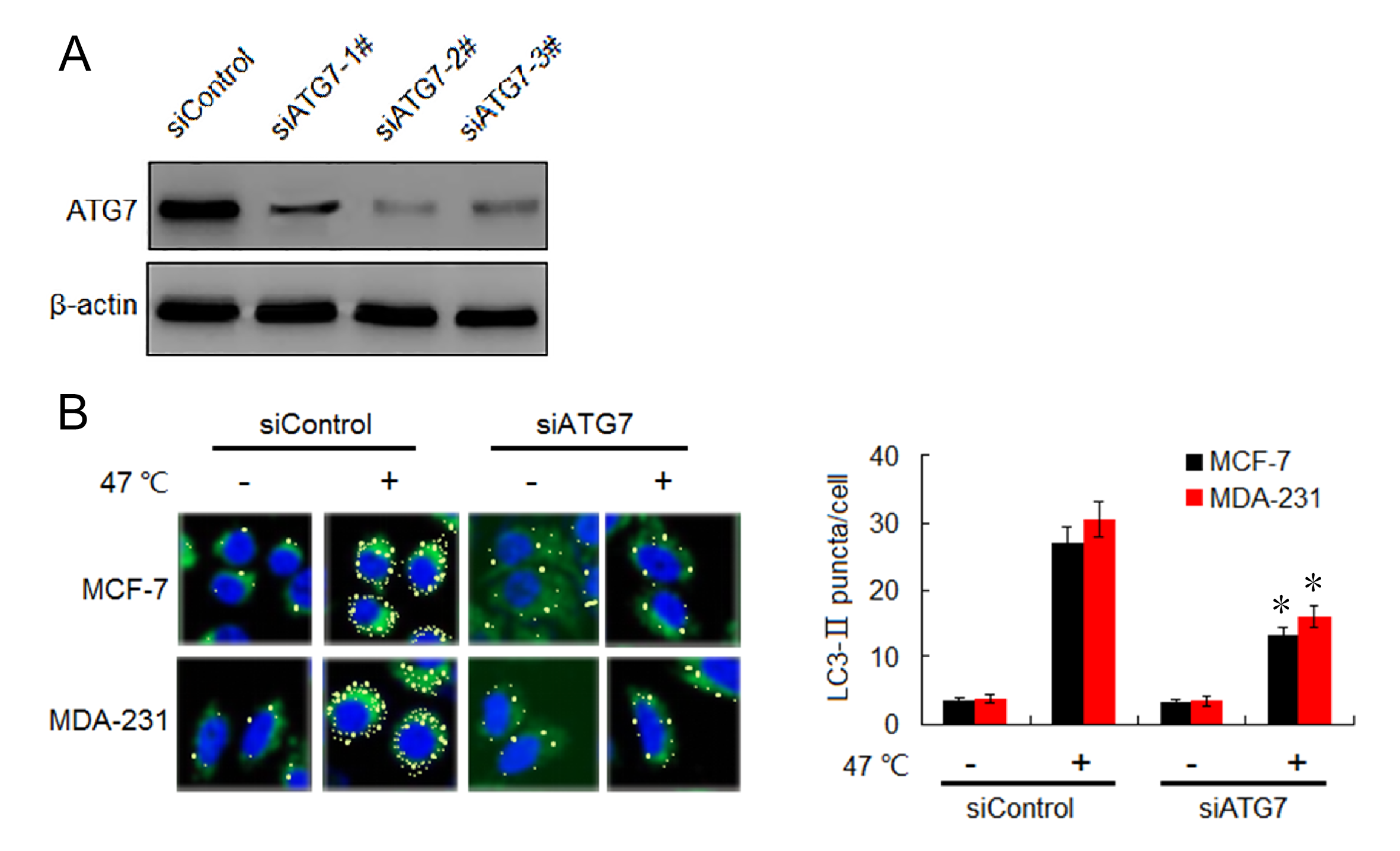

Supplement: Supplemental Information 1 — (A) ATG7 expressions in MDA-231 cells were analyzed by western blotting after transfection with siControl, siATG7-1#, siATG7-# and siATG7-3#. (B) After transfection with siATG7 or siControl, MCF-7 and MDA-231 cells were exposed to 47 °C for 30 min, or maintained at 37 °C, followed by culture at 37 °C for 24 h, then the LC3-II puncta formation was detected using immunofluorescent analysis and imaged by confocal microscope (left). The number of LC3-II puncta/cell was quantified by Image-Pro plus 5.1 software (right) (∗P < 0.01 vs 47 °C + siControl). [file peerj-11-14640-s001.png]

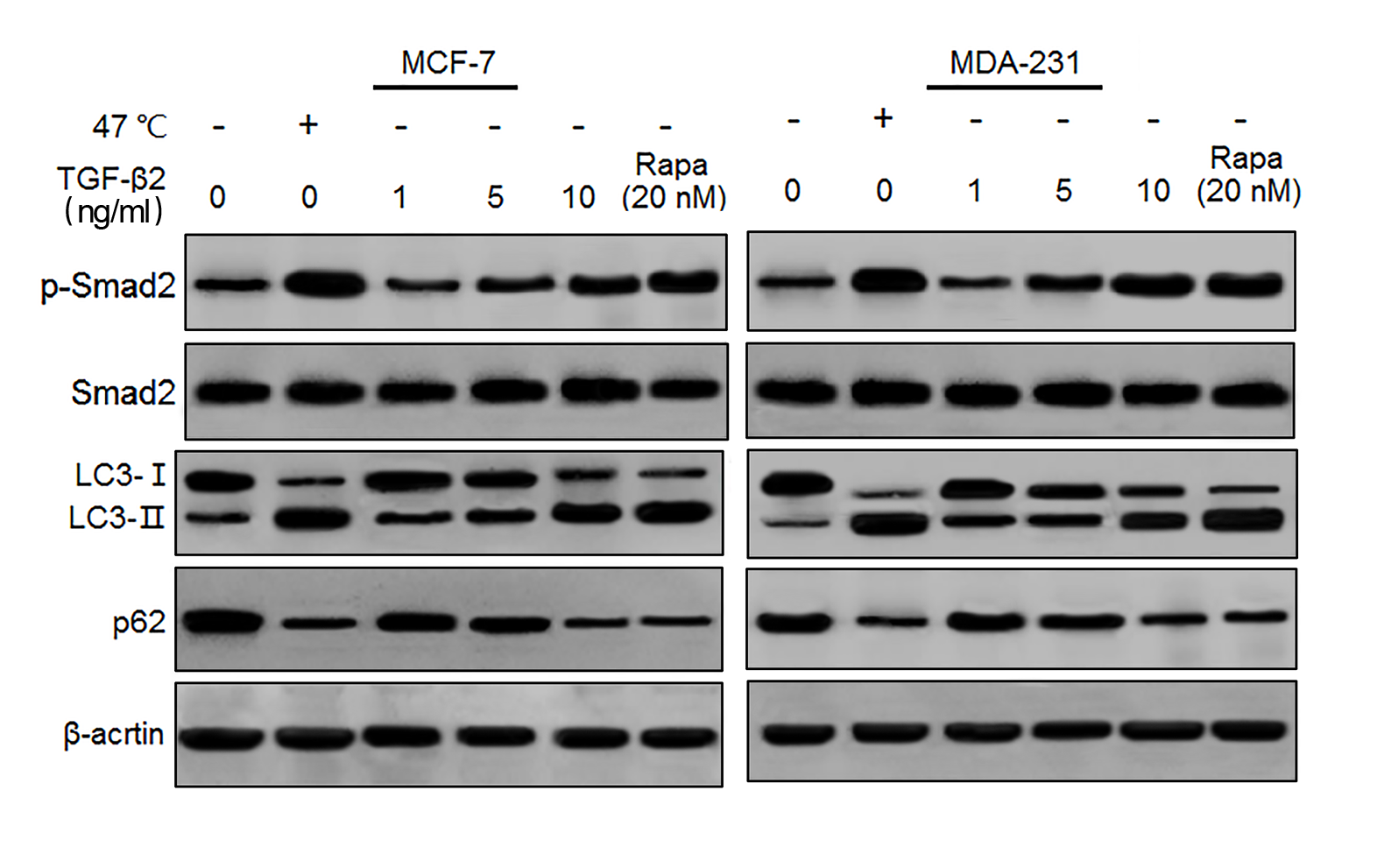

Supplement: Supplemental Information 2 — MCF-7 and MDA-231 cells were exposed to 47 °C for 30 min, followed by culture at 37 °C for 24 h, or treated with a TGF- β2 dose gradient ranging from 1 ng/ml to 10 ng/ml for 24 h, or treated with rapamycin (20 nM) for 24 h, The expressions of the indicated proteins were detected by western blotting. [file peerj-11-14640-s002.png]

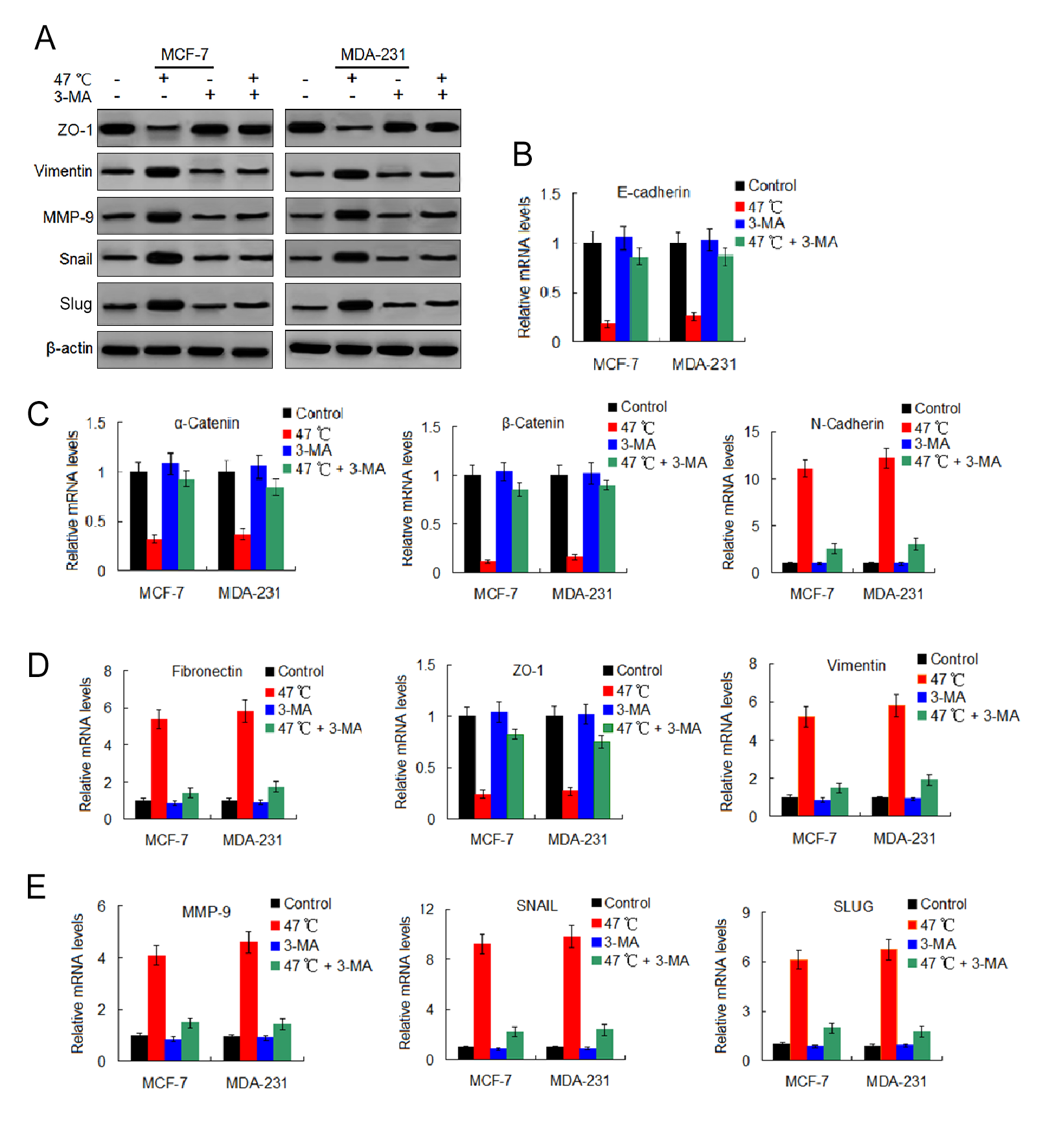

Supplement: Supplemental Information 3 — (A) MCF-7 and MDA-231 cells were exposed to 47 °C for 30 min in the absence or presence of 3-MA (10 µM), followed culture at 37 °C for 24 h. The expressions of the indicated proteins were detected by western blotting. (B–E) MCF-7 and MDA-231 cells were treated as (A), relative levels of the indicated miRNAs were analyzed by real-time quantitative PCR. [file peerj-11-14640-s003.png]

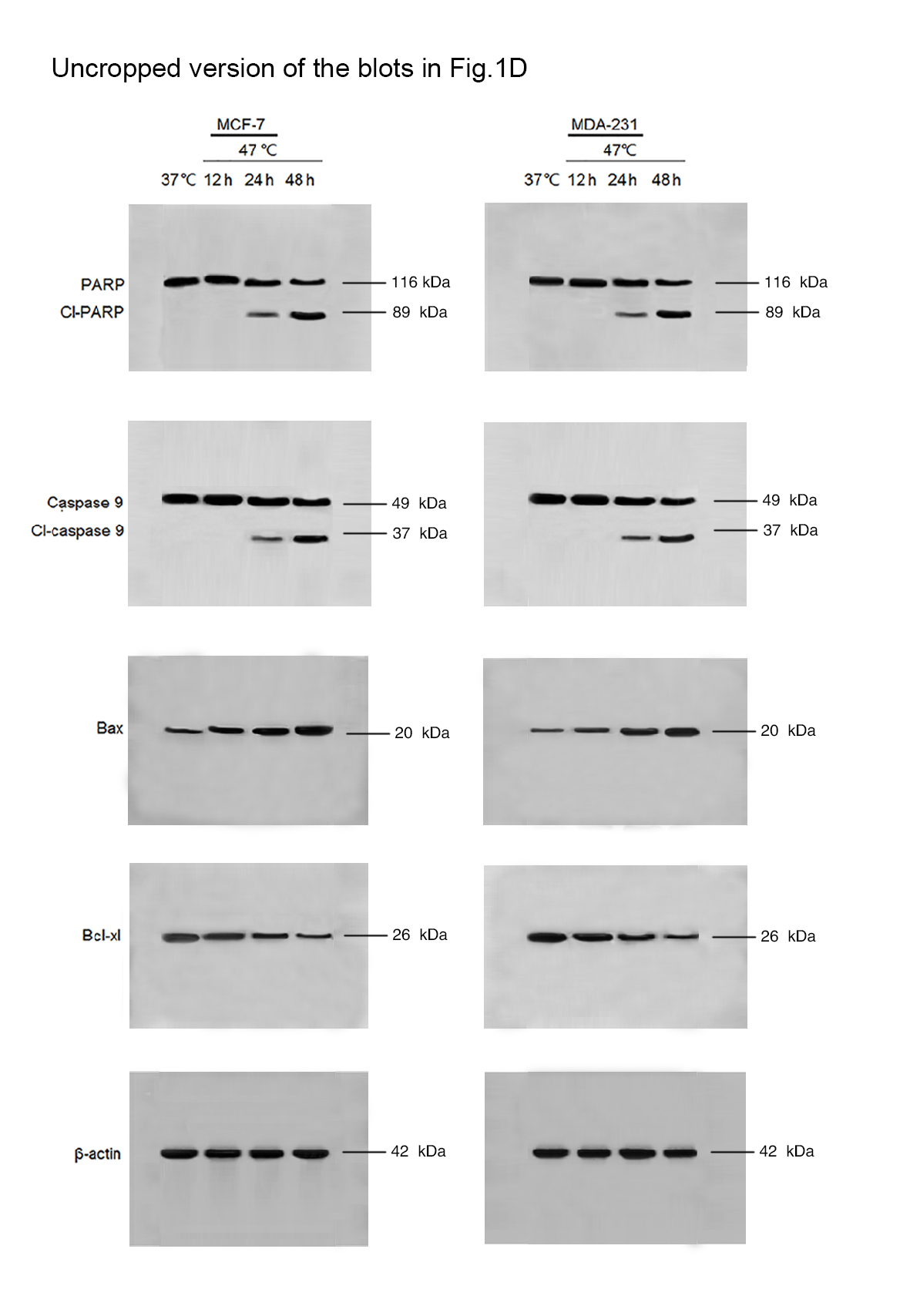

Supplement: Supplemental Information 6 — Raw data of Western blotting include Fig. 1–Fig. 3, Figs. 5 and 6. [file peerj-11-14640-s006.zip › Raw data of WB/Raw data of WB in Figure 1/Figure 1D.png]

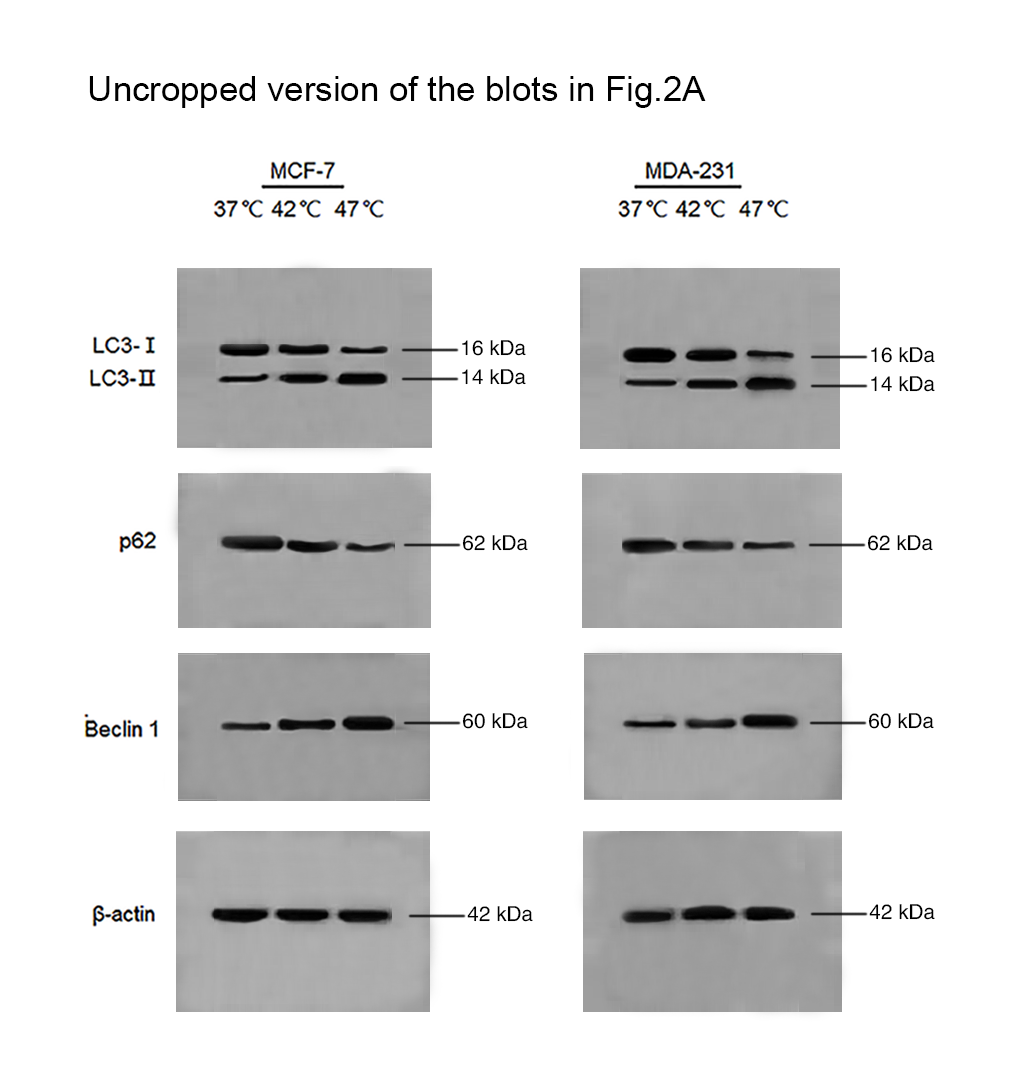

Supplement: Supplemental Information 6 — Raw data of Western blotting include Fig. 1–Fig. 3, Figs. 5 and 6. [file peerj-11-14640-s006.zip › Raw data of WB/Raw data of WB in Figure 2/Figure 2A.png]

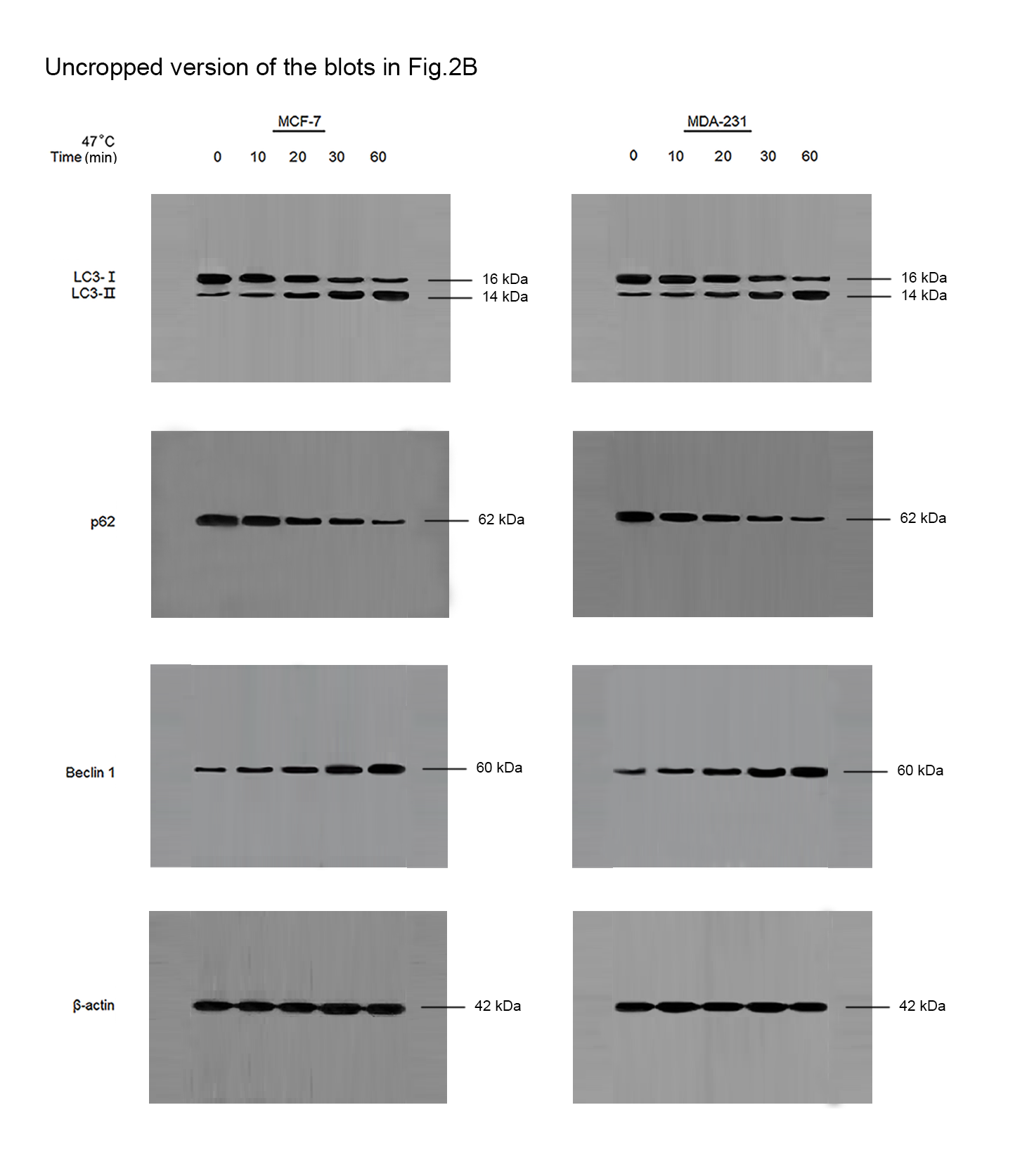

Supplement: Supplemental Information 6 — Raw data of Western blotting include Fig. 1–Fig. 3, Figs. 5 and 6. [file peerj-11-14640-s006.zip › Raw data of WB/Raw data of WB in Figure 2/Figure 2B.png]

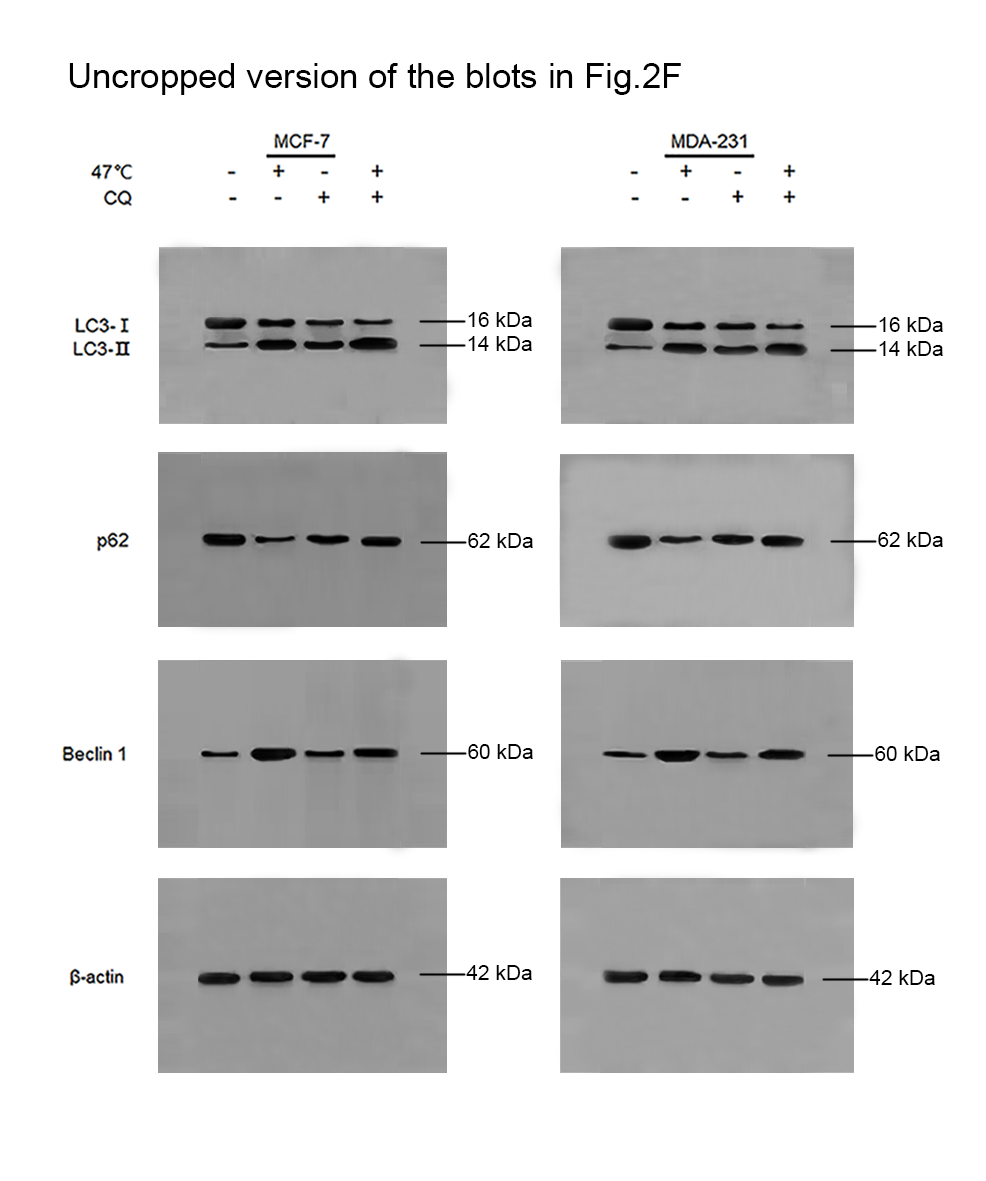

Supplement: Supplemental Information 6 — Raw data of Western blotting include Fig. 1–Fig. 3, Figs. 5 and 6. [file peerj-11-14640-s006.zip › Raw data of WB/Raw data of WB in Figure 2/Figure 2F.png]

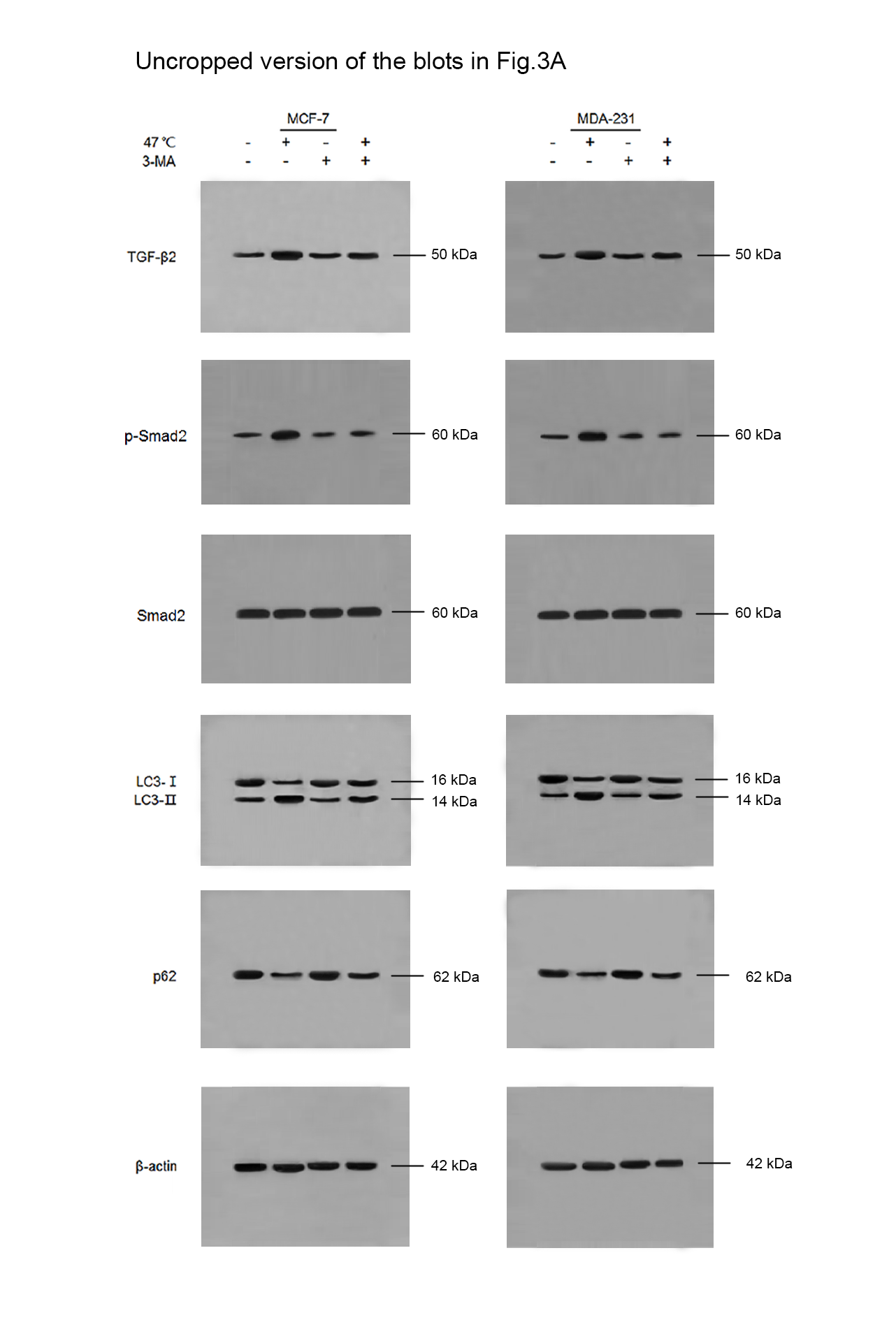

Supplement: Supplemental Information 6 — Raw data of Western blotting include Fig. 1–Fig. 3, Figs. 5 and 6. [file peerj-11-14640-s006.zip › Raw data of WB/Raw data of WB in Figure 3/Figure 3A.png]

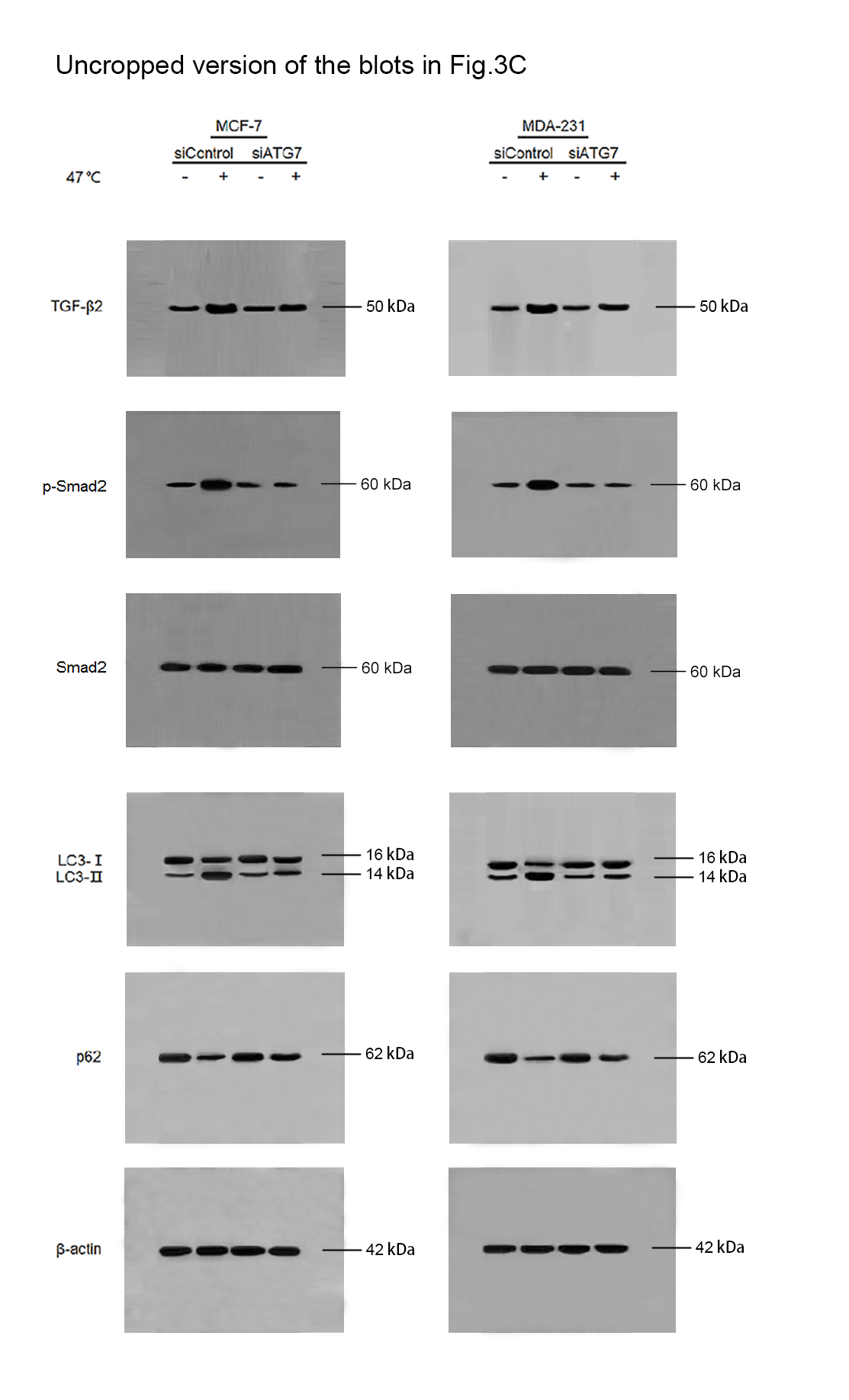

Supplement: Supplemental Information 6 — Raw data of Western blotting include Fig. 1–Fig. 3, Figs. 5 and 6. [file peerj-11-14640-s006.zip › Raw data of WB/Raw data of WB in Figure 3/Figure 3C.png]

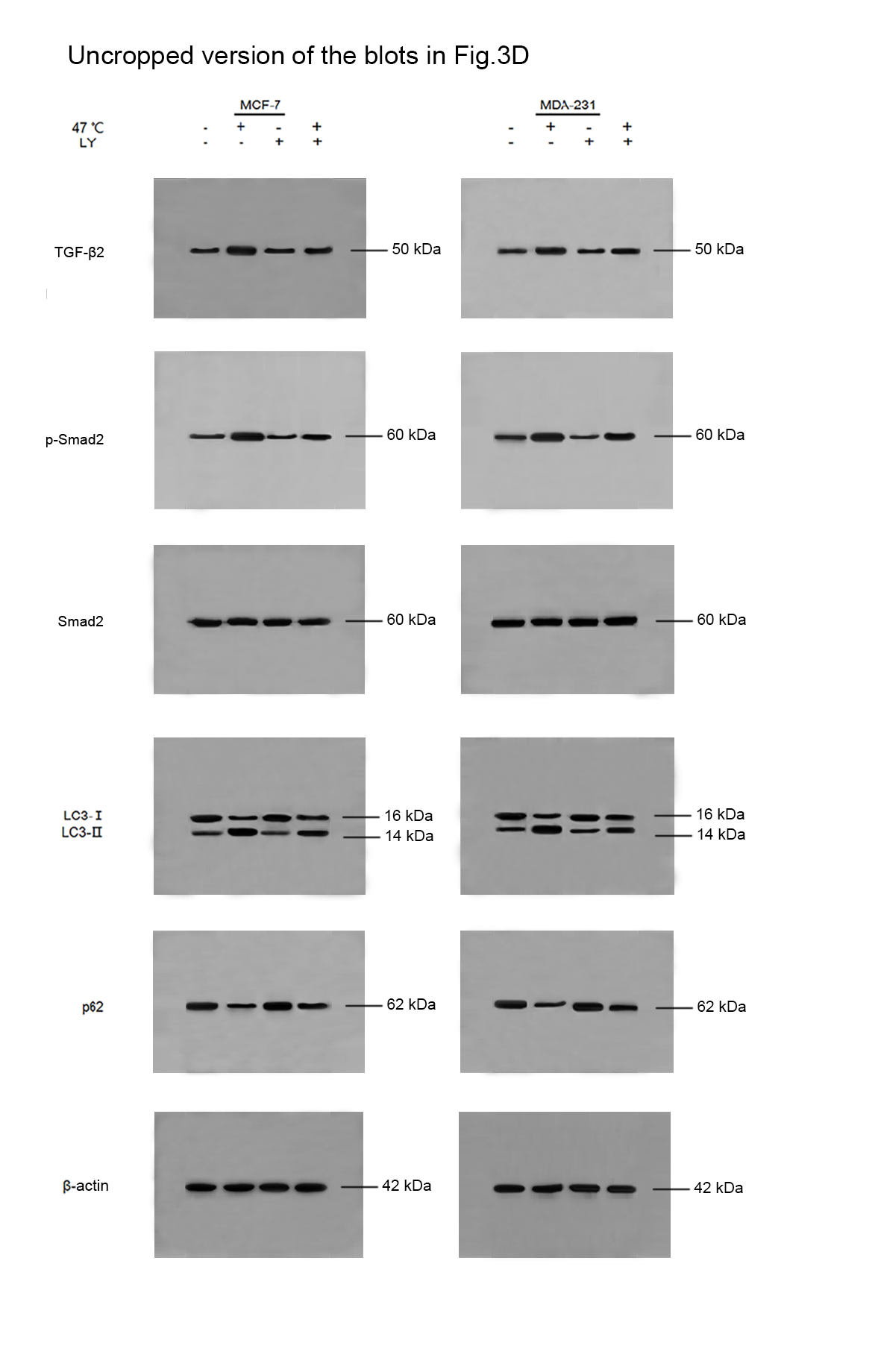

Supplement: Supplemental Information 6 — Raw data of Western blotting include Fig. 1–Fig. 3, Figs. 5 and 6. [file peerj-11-14640-s006.zip › Raw data of WB/Raw data of WB in Figure 3/Figure 3D.png]

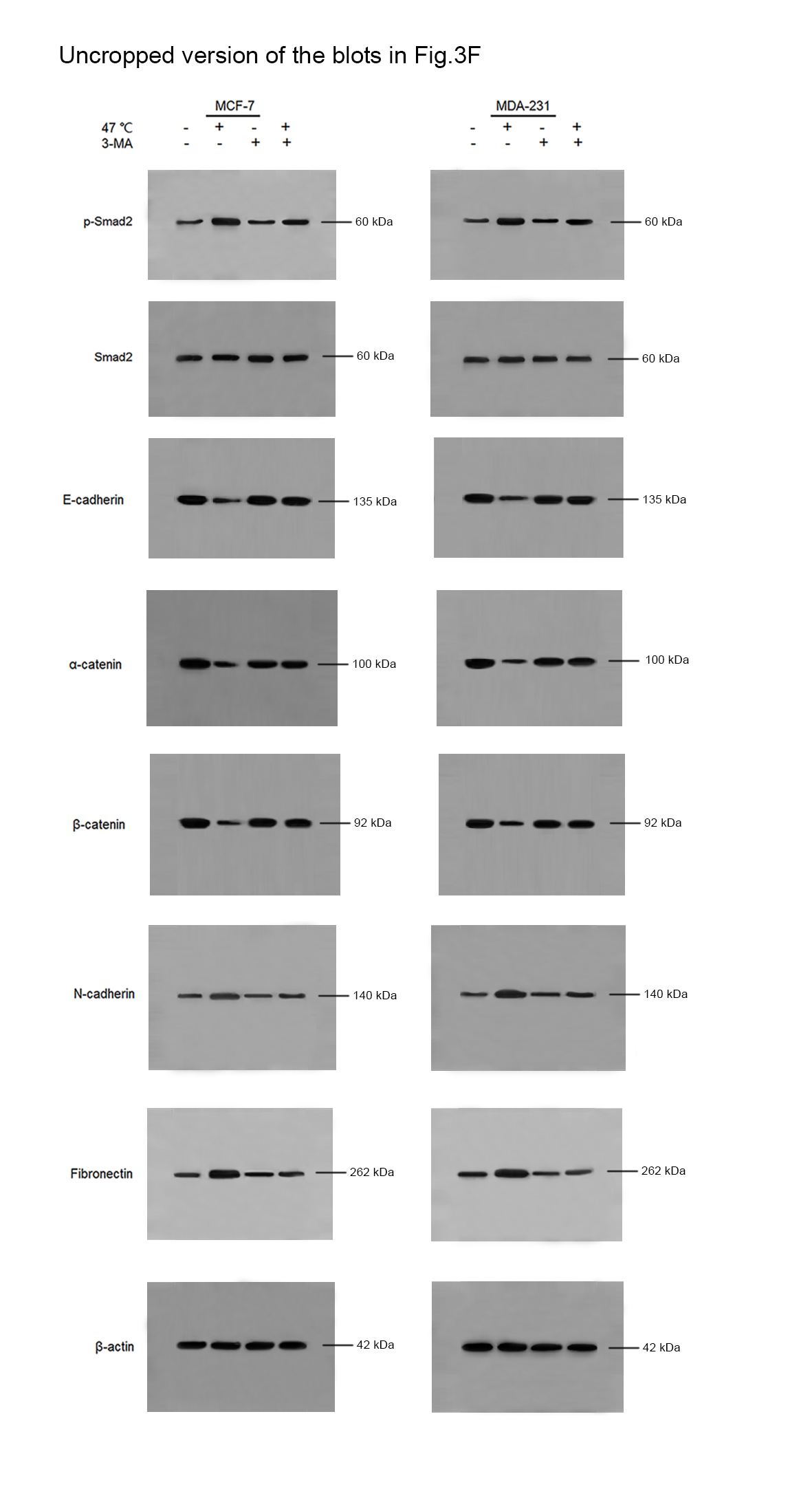

Supplement: Supplemental Information 6 — Raw data of Western blotting include Fig. 1–Fig. 3, Figs. 5 and 6. [file peerj-11-14640-s006.zip › Raw data of WB/Raw data of WB in Figure 3/Figure 3F.png]

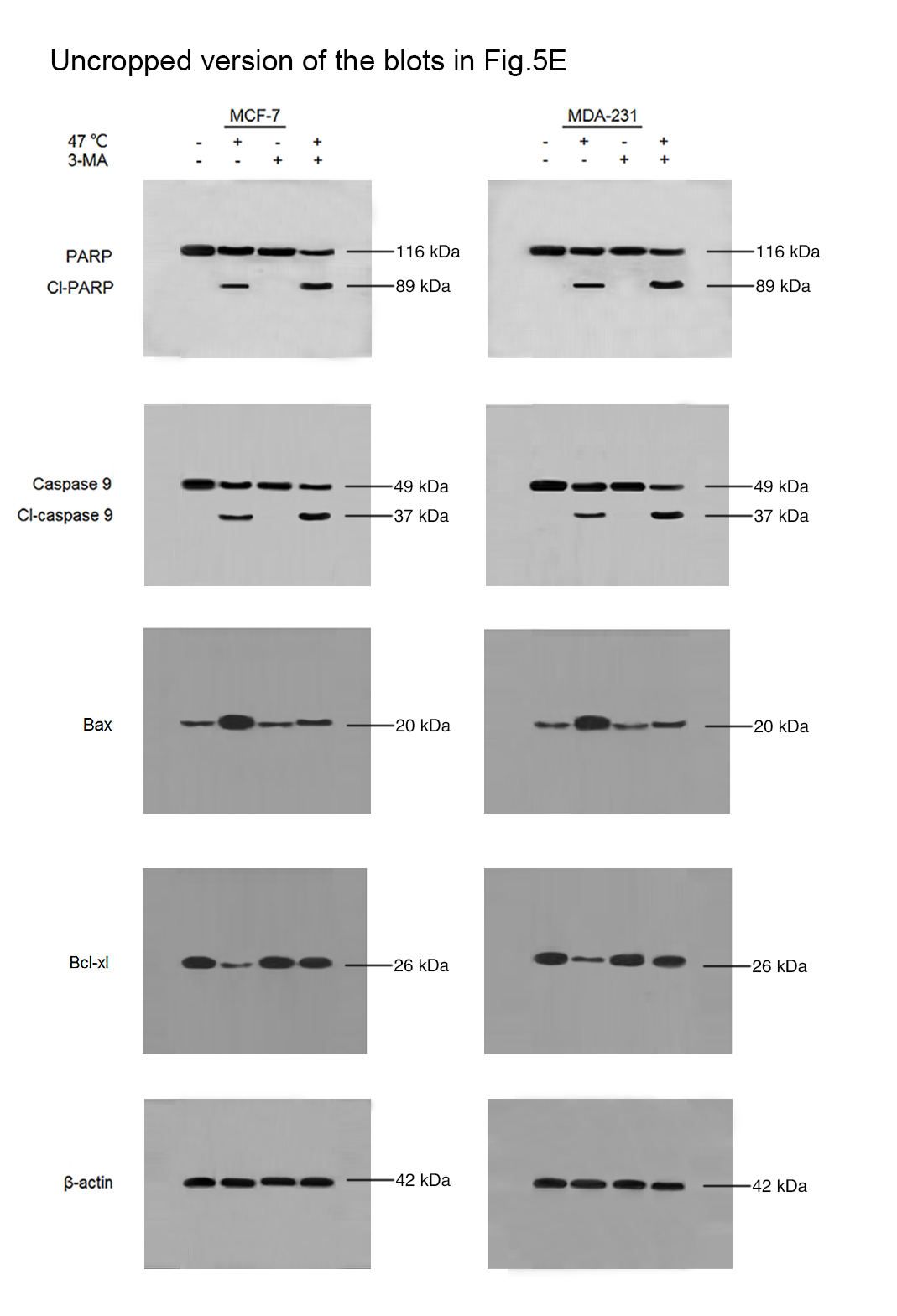

Supplement: Supplemental Information 6 — Raw data of Western blotting include Fig. 1–Fig. 3, Figs. 5 and 6. [file peerj-11-14640-s006.zip › Raw data of WB/Raw data of WB in Figure 5/Figure 5E.png]

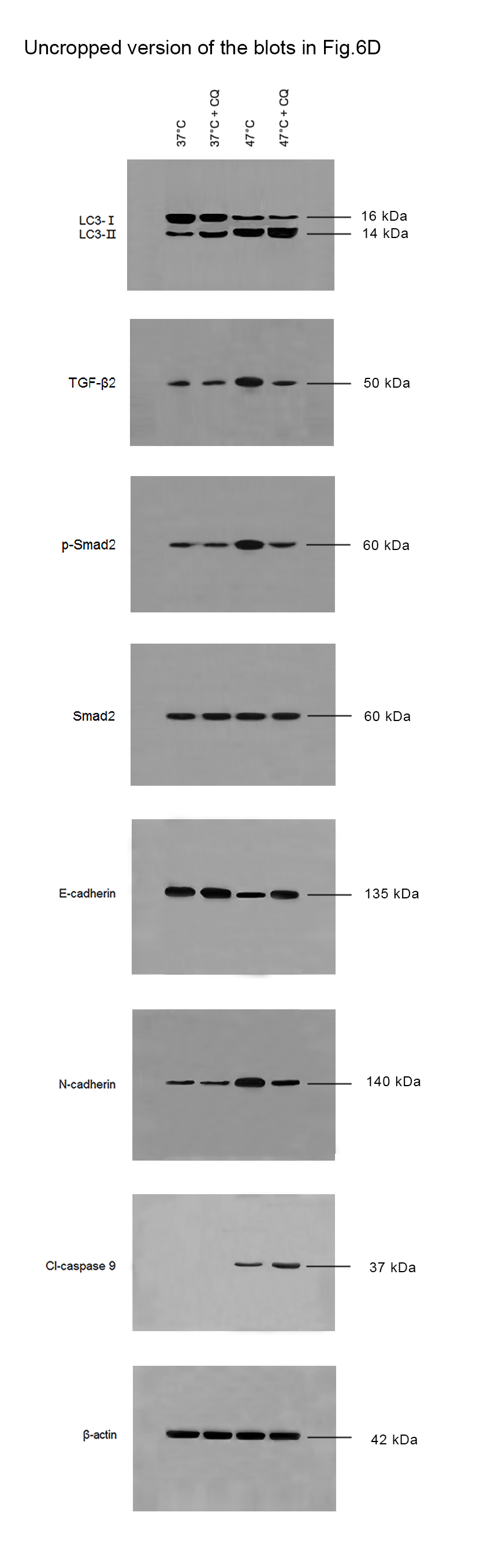

Supplement: Supplemental Information 6 — Raw data of Western blotting include Fig. 1–Fig. 3, Figs. 5 and 6. [file peerj-11-14640-s006.zip › Raw data of WB/Raw data of WB in Figure 6/Figure 6 .png]

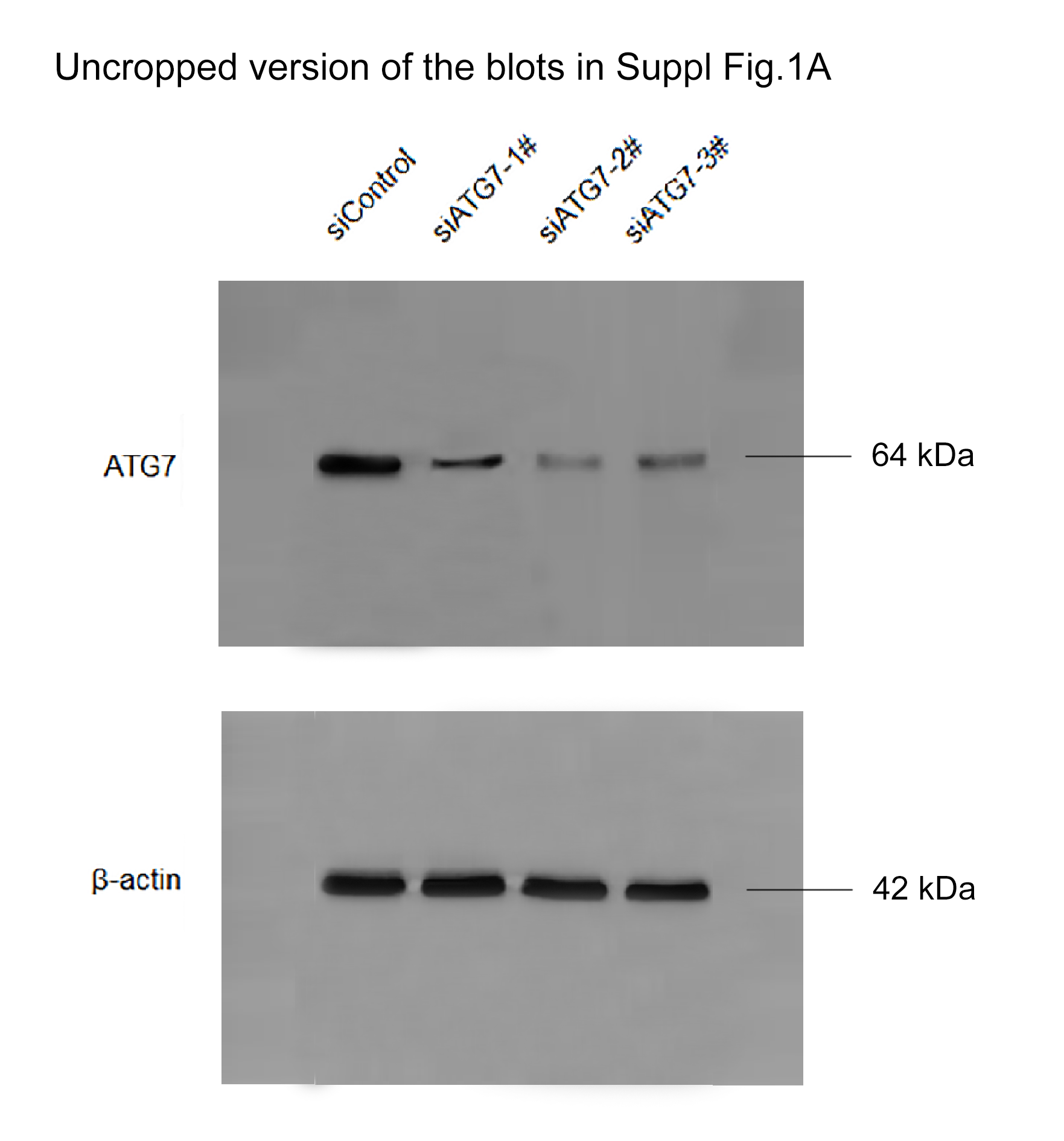

Supplement: Supplemental Information 6 — Raw data of Western blotting include Fig. 1–Fig. 3, Figs. 5 and 6. [file peerj-11-14640-s006.zip › Raw data of WB/Raw data of WB in supplementary Figures/Suppl Figure 1A.png]

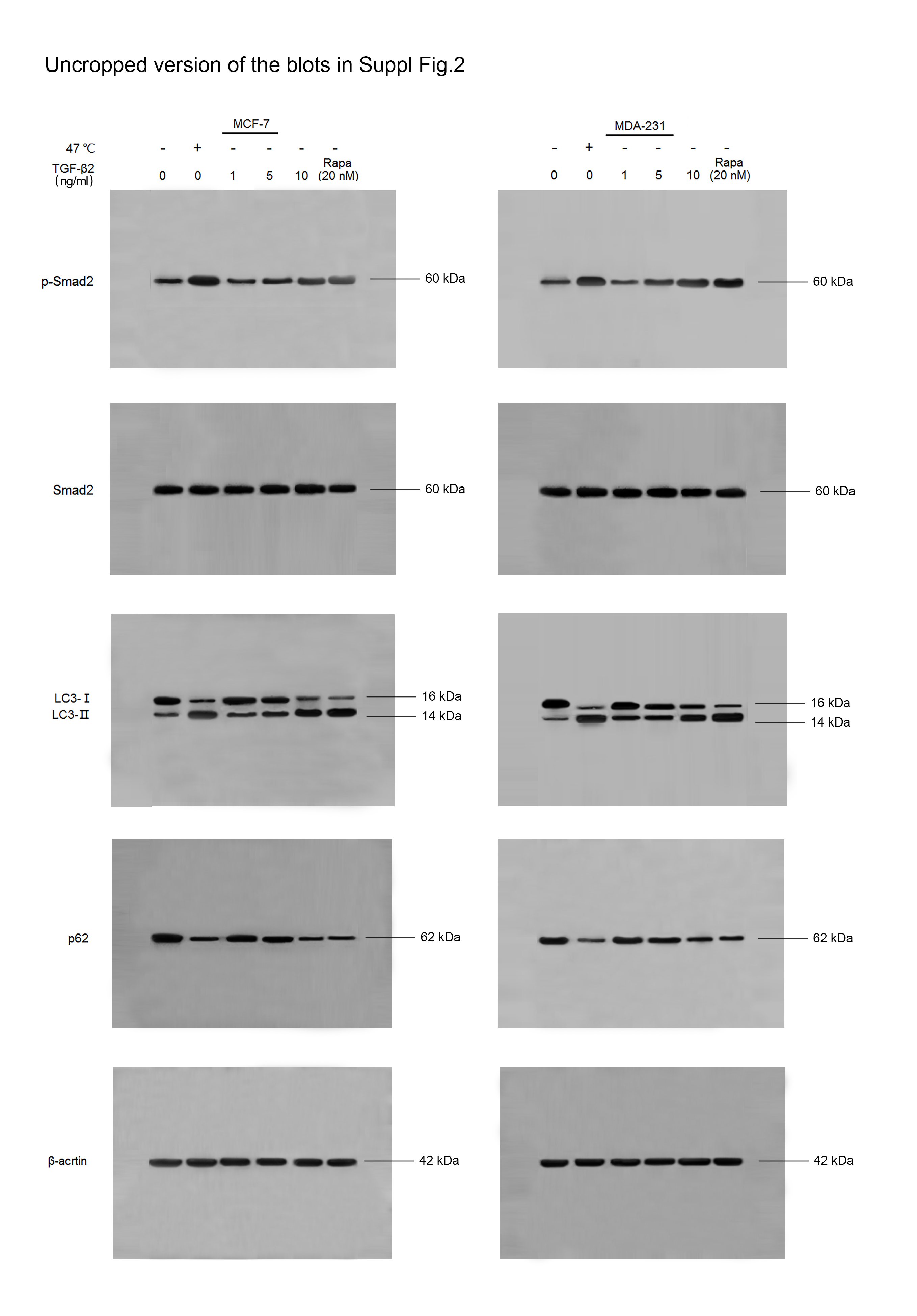

Supplement: Supplemental Information 6 — Raw data of Western blotting include Fig. 1–Fig. 3, Figs. 5 and 6. [file peerj-11-14640-s006.zip › Raw data of WB/Raw data of WB in supplementary Figures/Suppl figure 2.png]

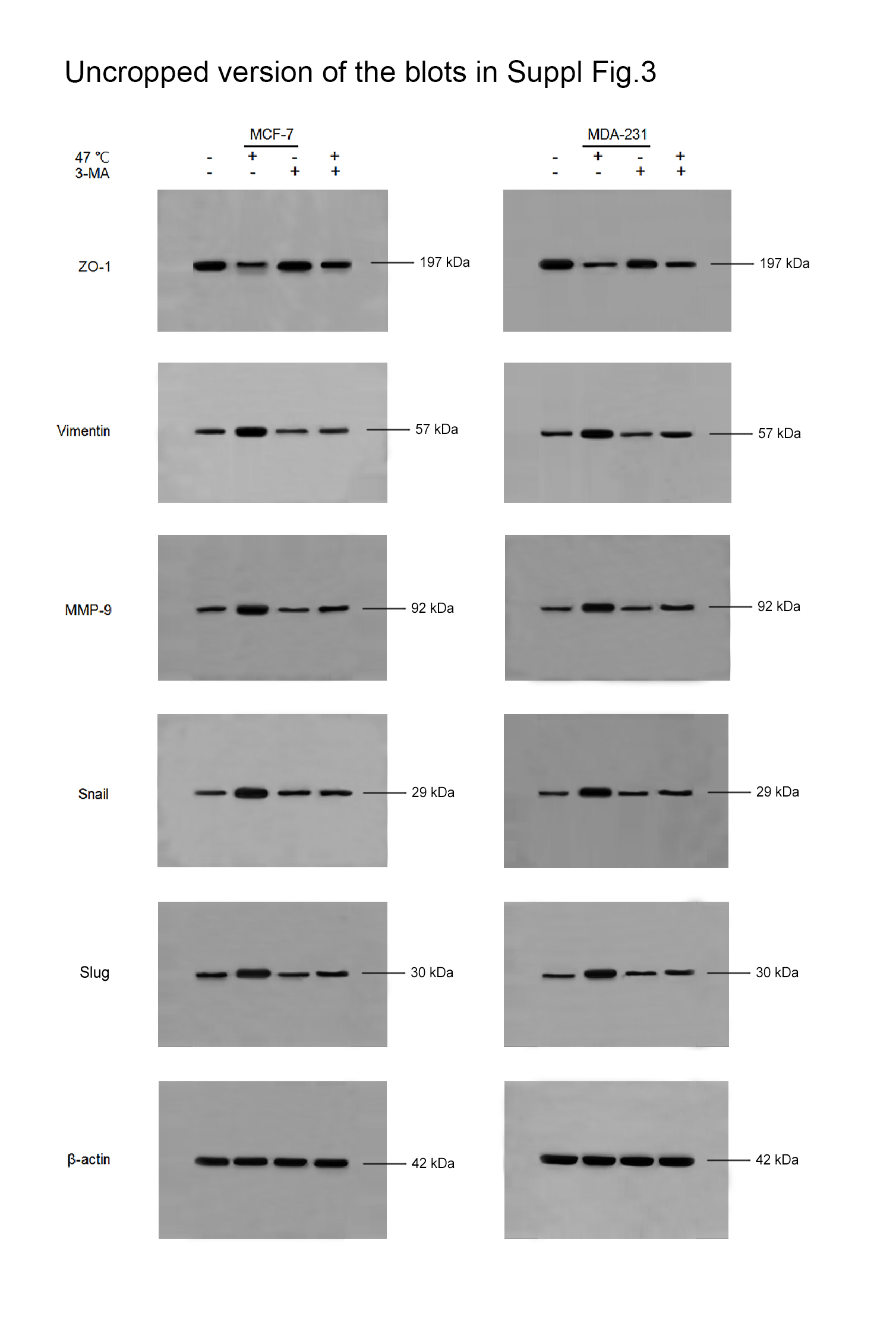

Supplement: Supplemental Information 6 — Raw data of Western blotting include Fig. 1–Fig. 3, Figs. 5 and 6. [file peerj-11-14640-s006.zip › Raw data of WB/Raw data of WB in supplementary Figures/Suppl Figure 3A.png]

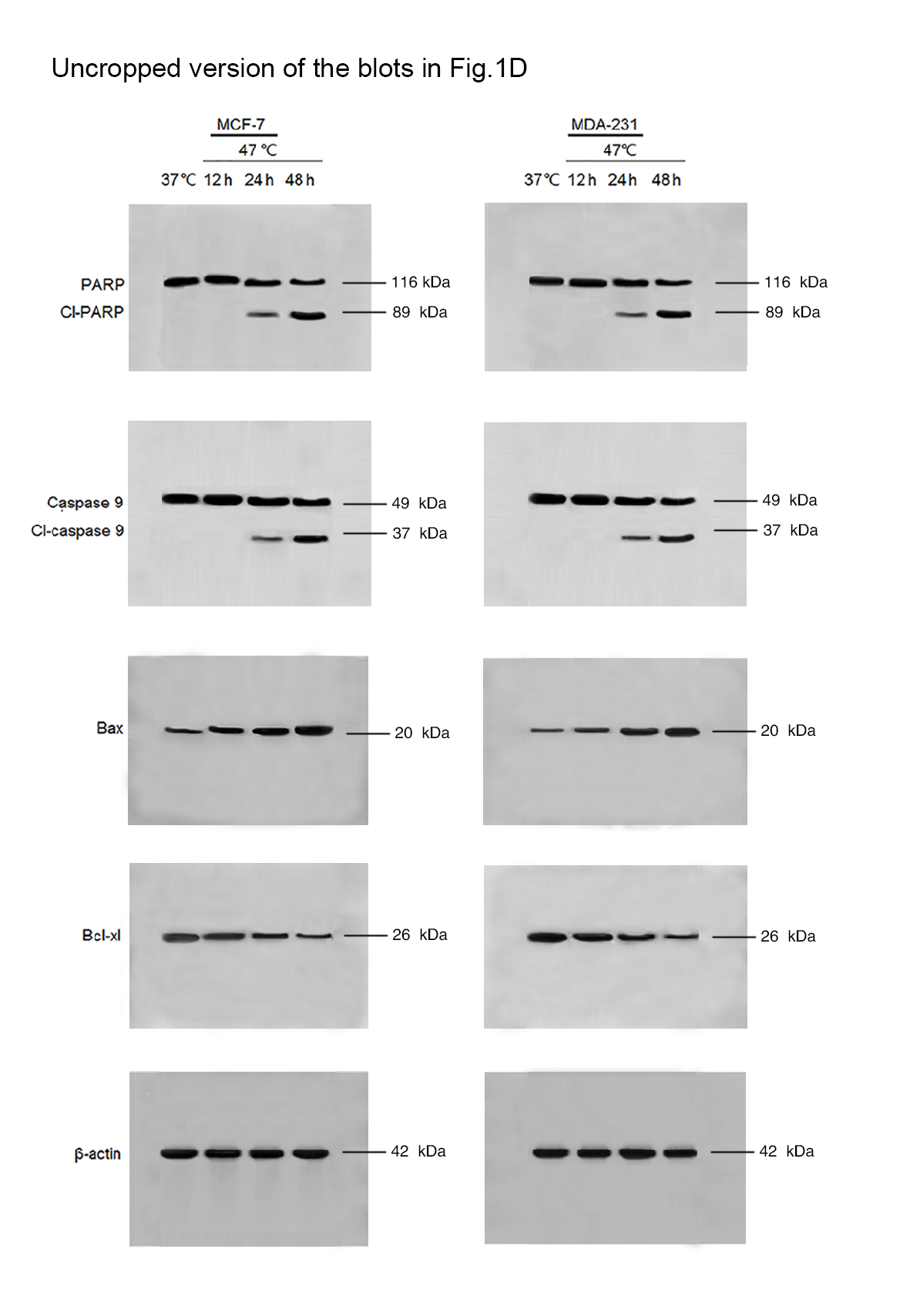

Supplement: Supplemental Information 6 — Raw data of Western blotting include Fig. 1–Fig. 3, Figs. 5 and 6. [file peerj-11-14640-s006.zip › Raw data of WB/Raw data of WB-TIF ╕±╩╜/Raw data of WB in Figure 1/Figure 1D.tif]

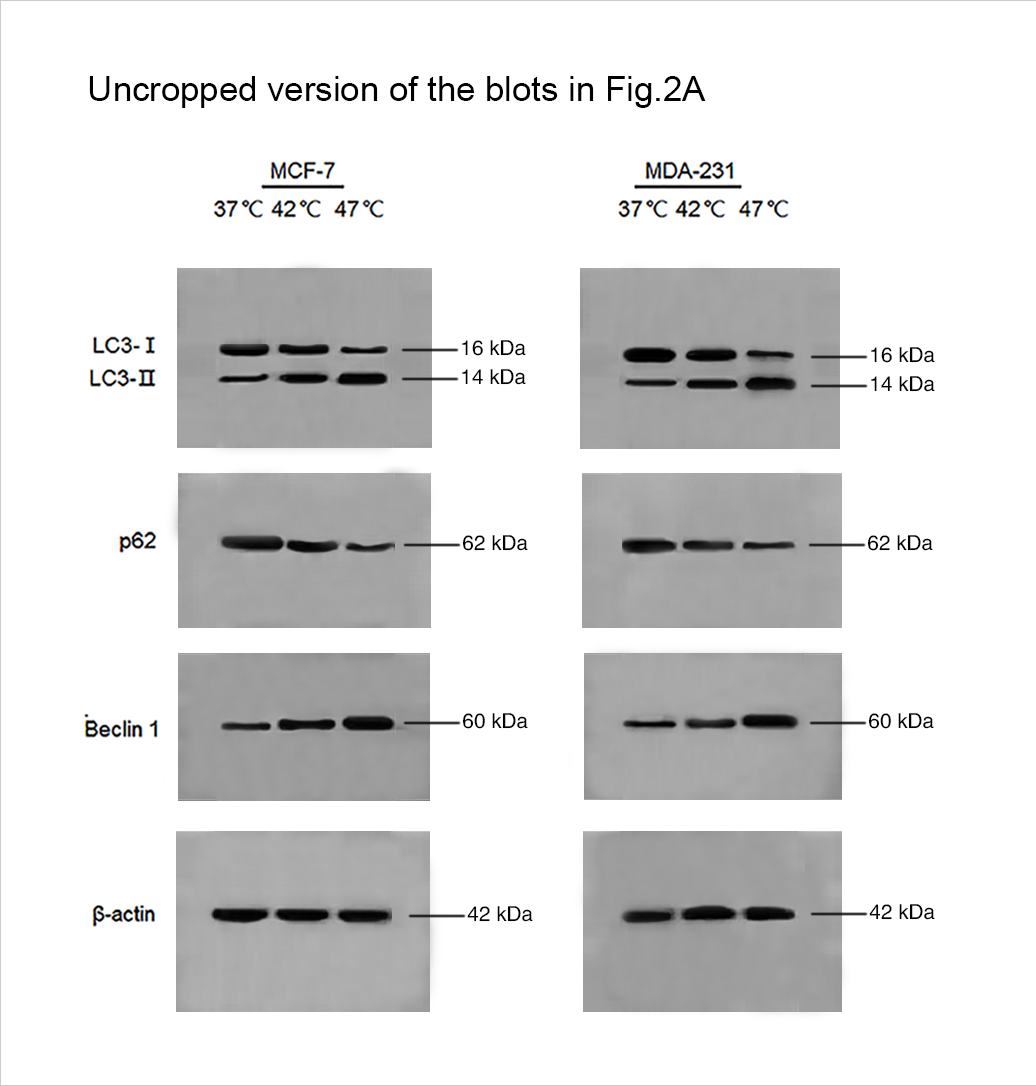

Supplement: Supplemental Information 6 — Raw data of Western blotting include Fig. 1–Fig. 3, Figs. 5 and 6. [file peerj-11-14640-s006.zip › Raw data of WB/Raw data of WB-TIF ╕±╩╜/Raw data of WB in Figure 2/Figure 2A.tif]

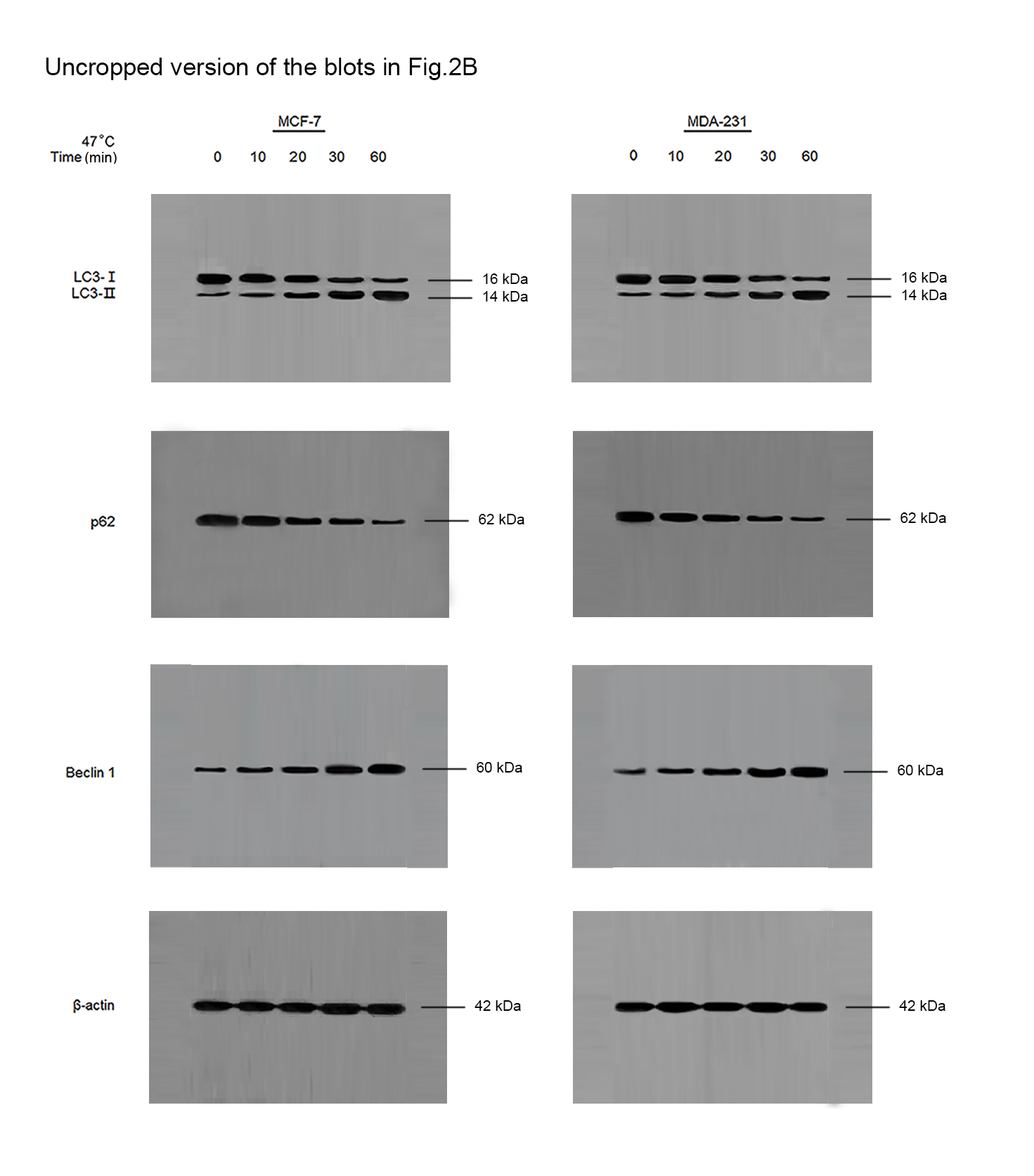

Supplement: Supplemental Information 6 — Raw data of Western blotting include Fig. 1–Fig. 3, Figs. 5 and 6. [file peerj-11-14640-s006.zip › Raw data of WB/Raw data of WB-TIF ╕±╩╜/Raw data of WB in Figure 2/Figure 2B.tif]

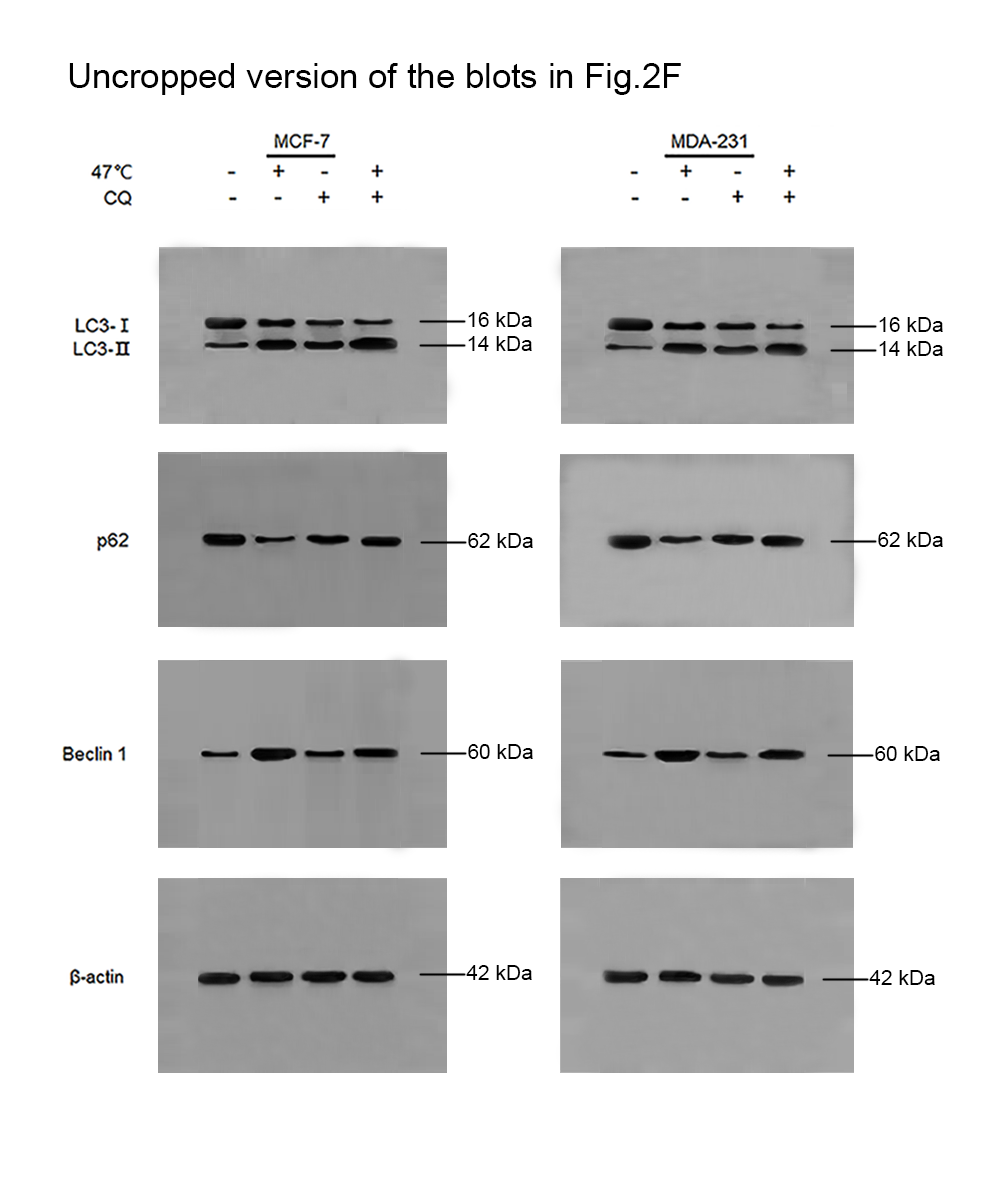

Supplement: Supplemental Information 6 — Raw data of Western blotting include Fig. 1–Fig. 3, Figs. 5 and 6. [file peerj-11-14640-s006.zip › Raw data of WB/Raw data of WB-TIF ╕±╩╜/Raw data of WB in Figure 2/Figure 2F.tif]

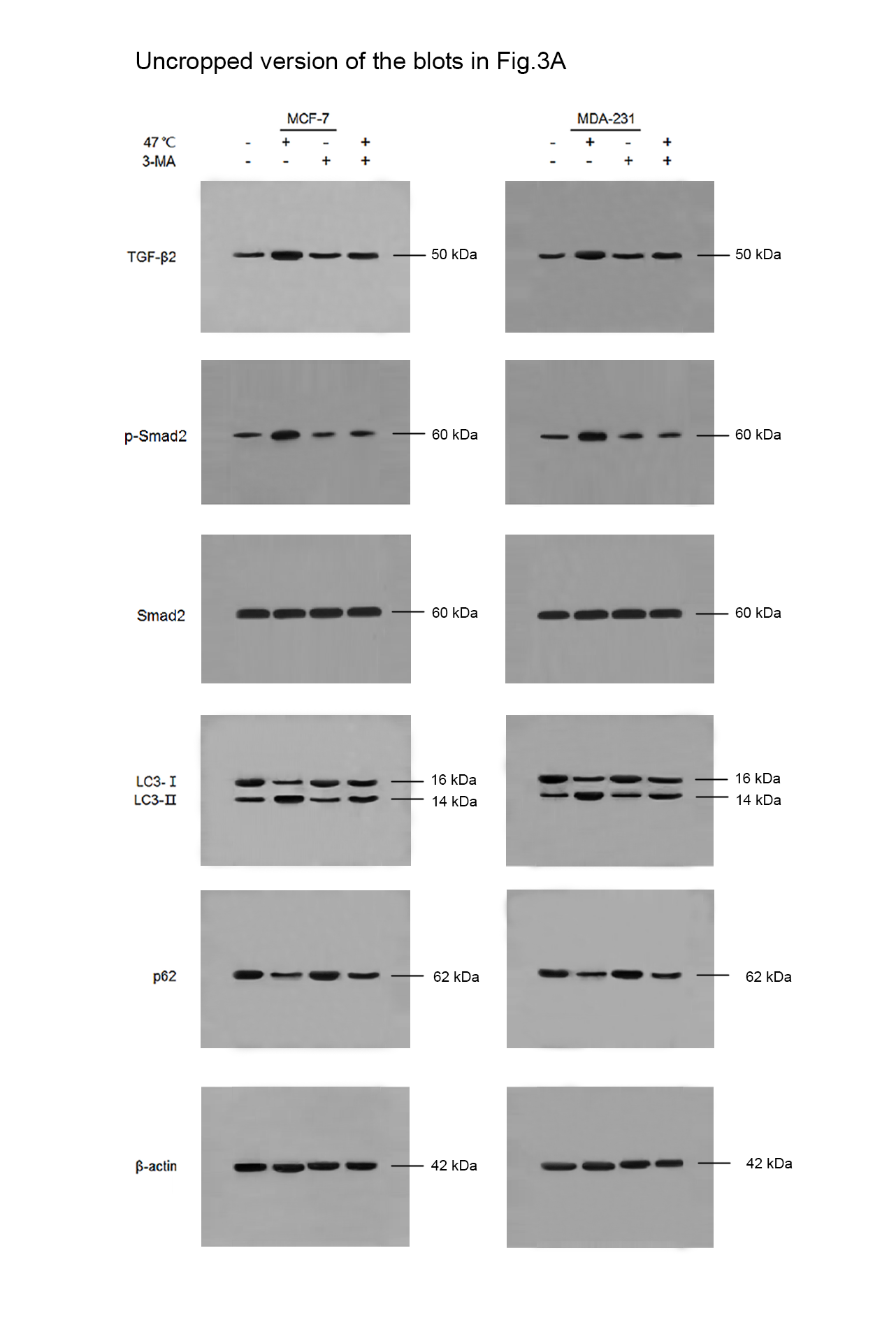

Supplement: Supplemental Information 6 — Raw data of Western blotting include Fig. 1–Fig. 3, Figs. 5 and 6. [file peerj-11-14640-s006.zip › Raw data of WB/Raw data of WB-TIF ╕±╩╜/Raw data of WB in Figure 3/Figure 3A.tif]

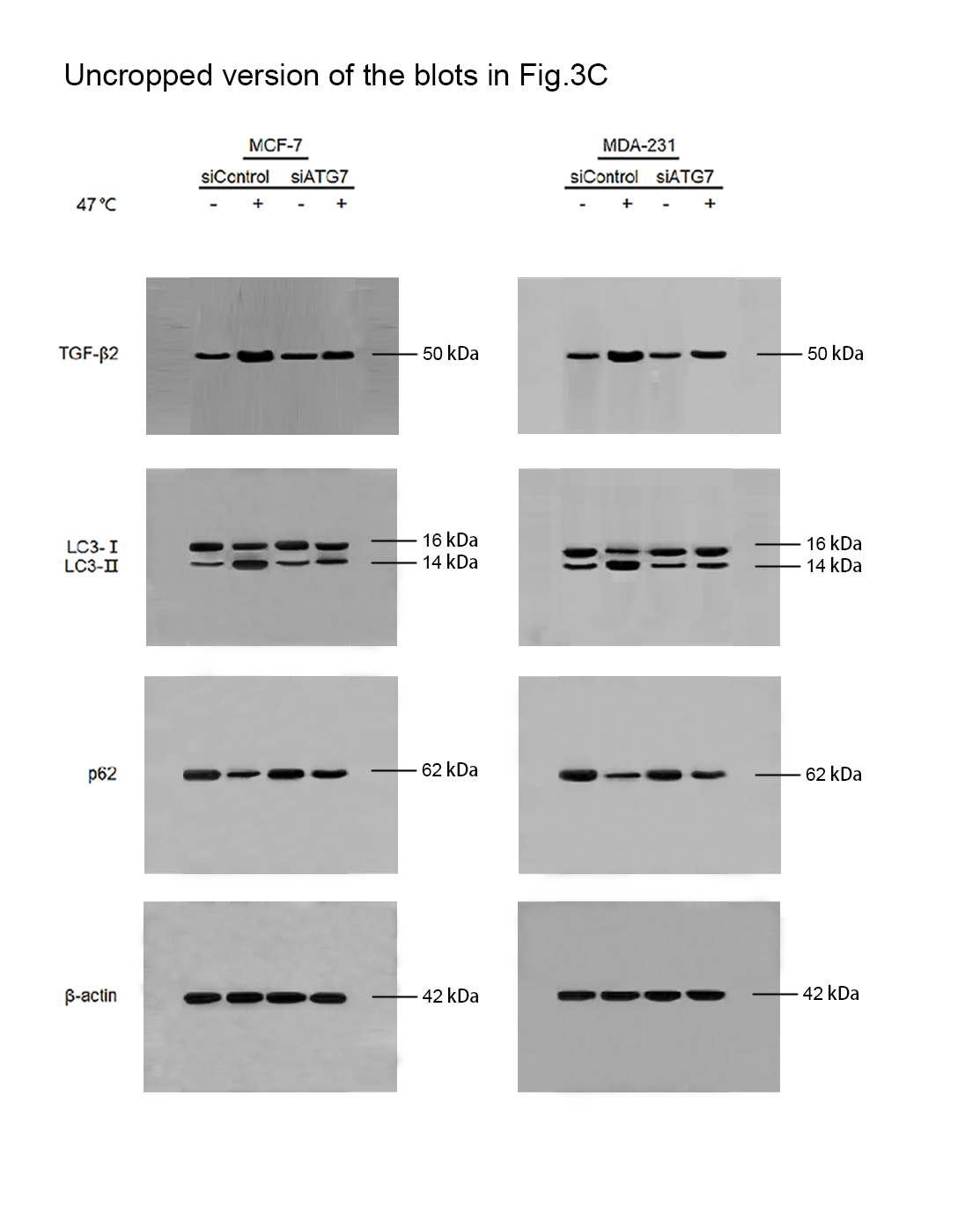

Supplement: Supplemental Information 6 — Raw data of Western blotting include Fig. 1–Fig. 3, Figs. 5 and 6. [file peerj-11-14640-s006.zip › Raw data of WB/Raw data of WB-TIF ╕±╩╜/Raw data of WB in Figure 3/Figure 3C.tif]

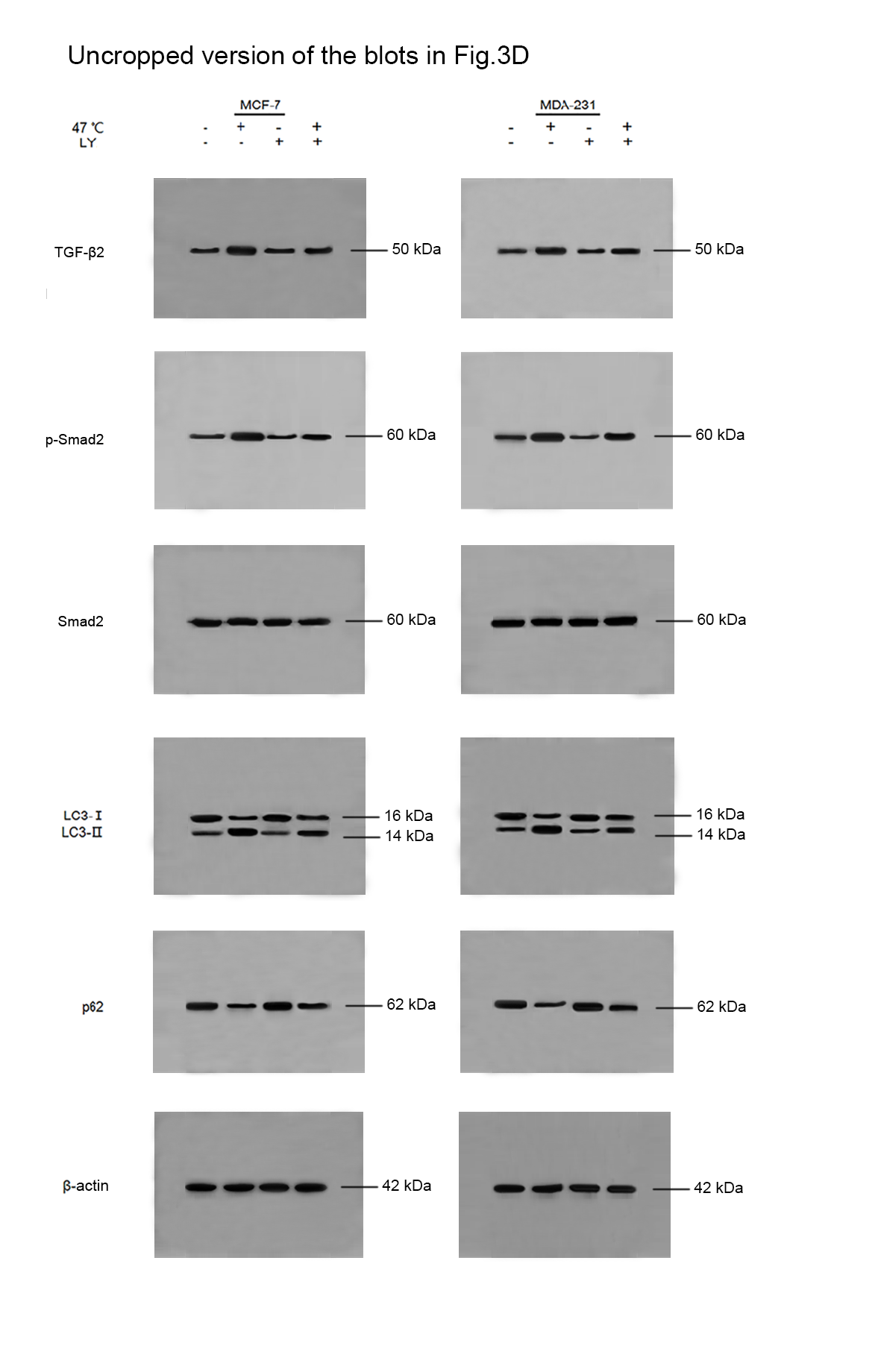

Supplement: Supplemental Information 6 — Raw data of Western blotting include Fig. 1–Fig. 3, Figs. 5 and 6. [file peerj-11-14640-s006.zip › Raw data of WB/Raw data of WB-TIF ╕±╩╜/Raw data of WB in Figure 3/Figure 3D.tif]

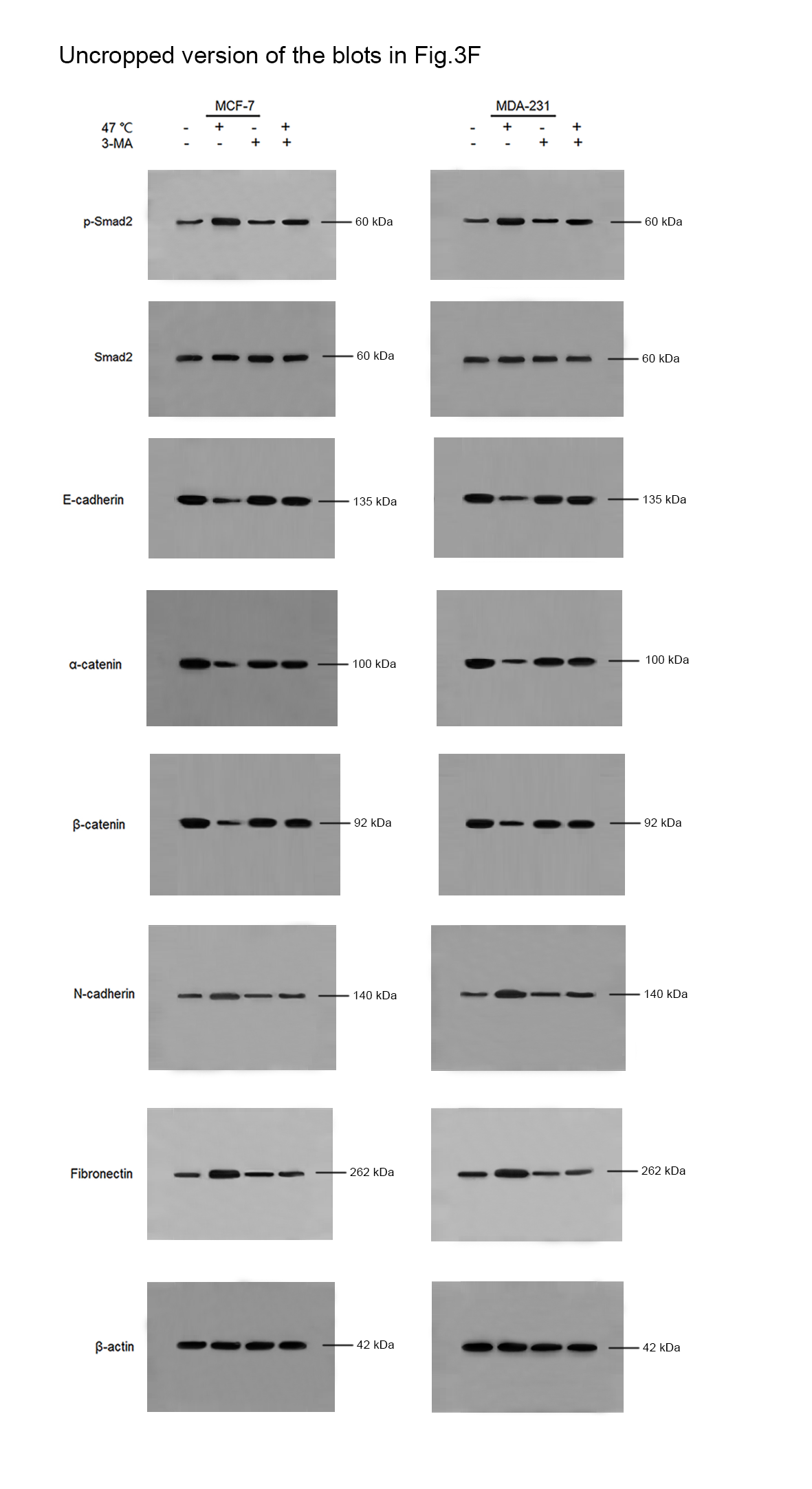

Supplement: Supplemental Information 6 — Raw data of Western blotting include Fig. 1–Fig. 3, Figs. 5 and 6. [file peerj-11-14640-s006.zip › Raw data of WB/Raw data of WB-TIF ╕±╩╜/Raw data of WB in Figure 3/Figure 3F.tif]

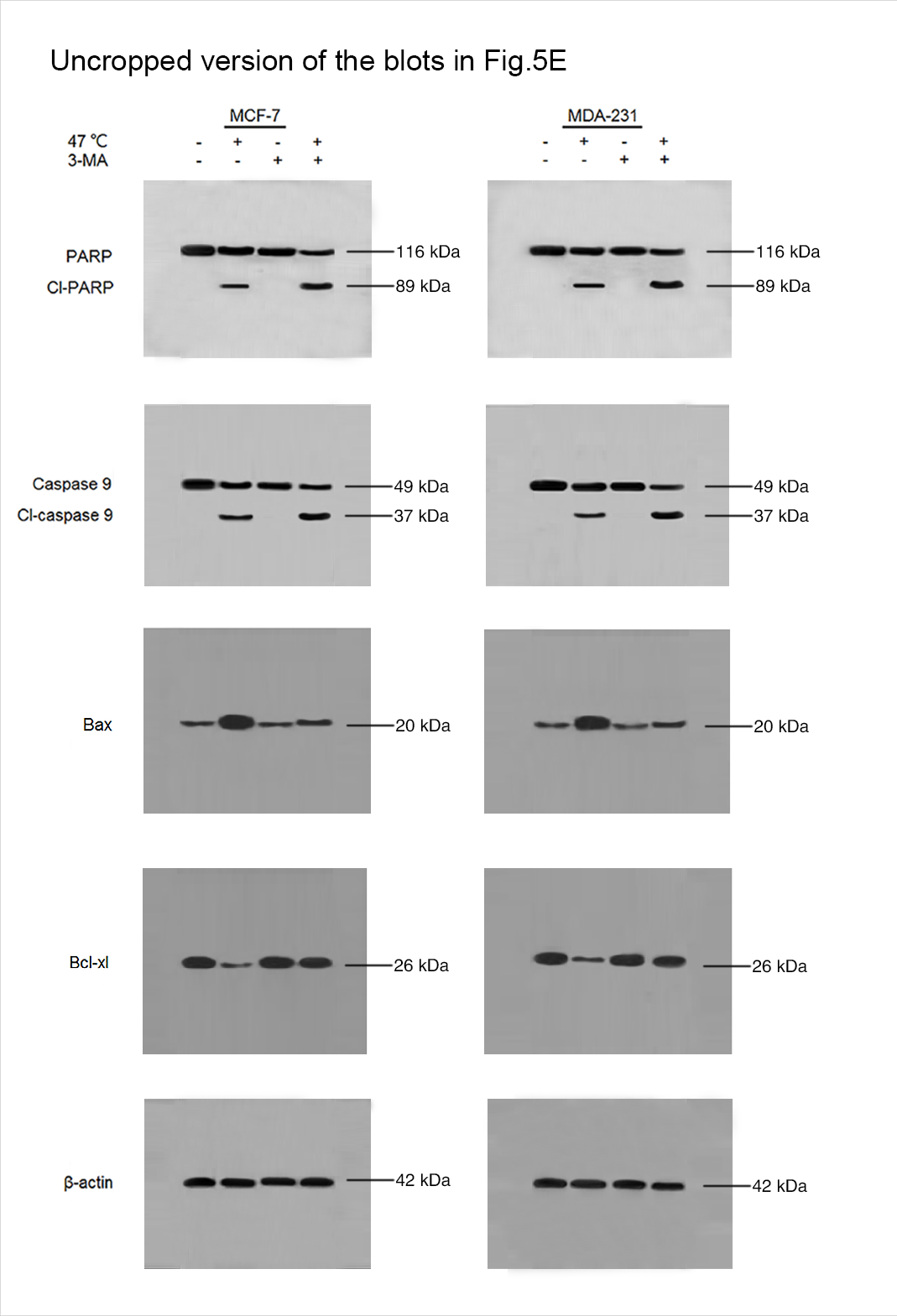

Supplement: Supplemental Information 6 — Raw data of Western blotting include Fig. 1–Fig. 3, Figs. 5 and 6. [file peerj-11-14640-s006.zip › Raw data of WB/Raw data of WB-TIF ╕±╩╜/Raw data of WB in Figure 5/Figure 5E.tif]

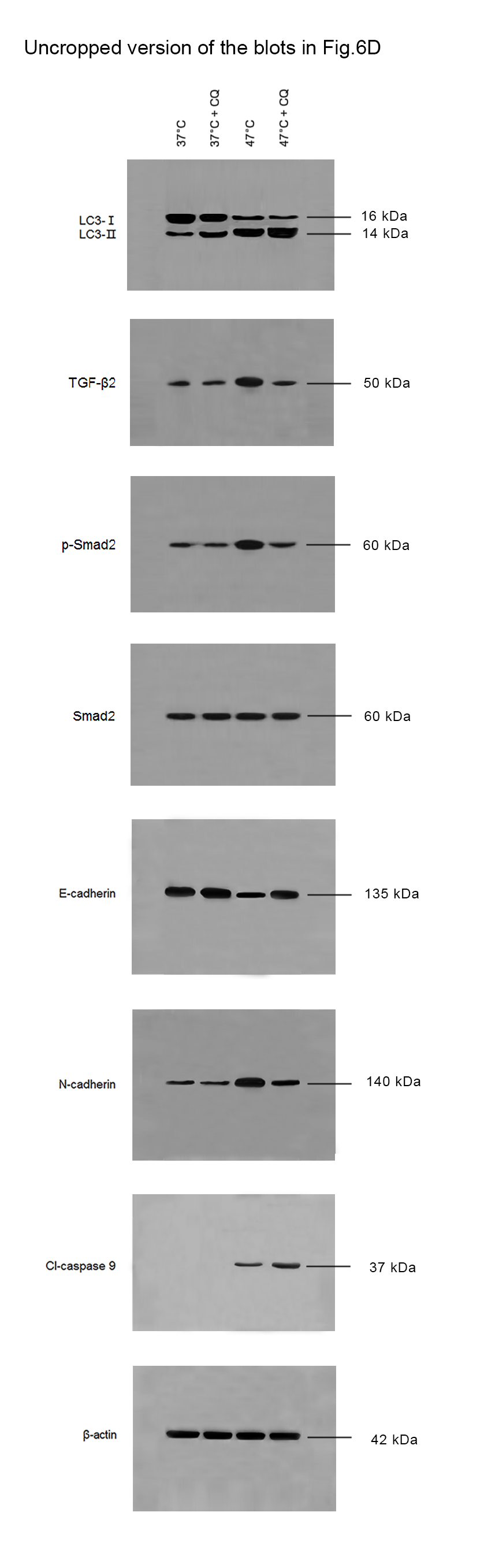

Supplement: Supplemental Information 6 — Raw data of Western blotting include Fig. 1–Fig. 3, Figs. 5 and 6. [file peerj-11-14640-s006.zip › Raw data of WB/Raw data of WB-TIF ╕±╩╜/Raw data of WB in Figure 6/Figure 6 .tif]

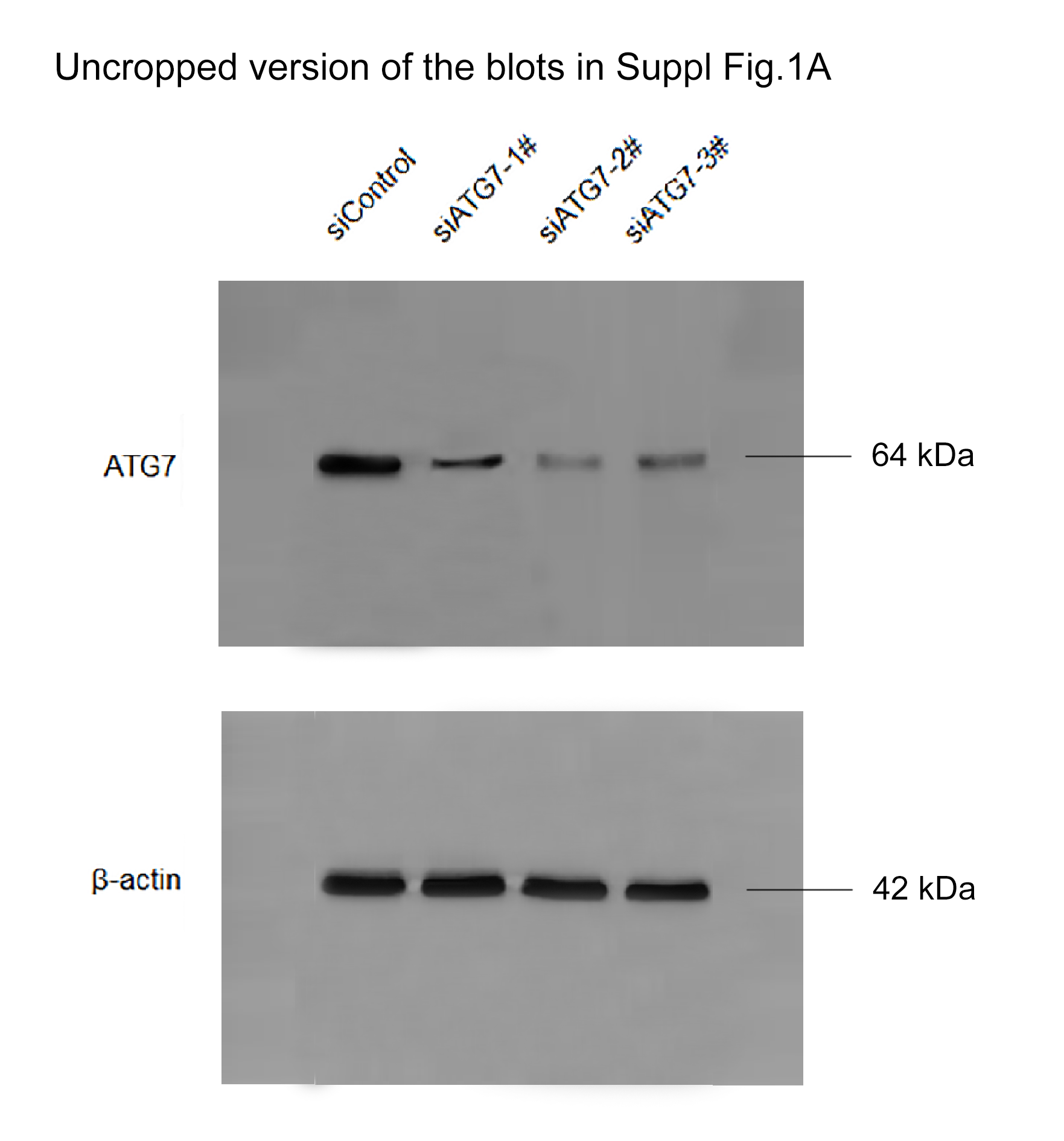

Supplement: Supplemental Information 6 — Raw data of Western blotting include Fig. 1–Fig. 3, Figs. 5 and 6. [file peerj-11-14640-s006.zip › Raw data of WB/Raw data of WB-TIF ╕±╩╜/Raw data of WB in supplementary Figures/Suppl Figure 1A.tif]

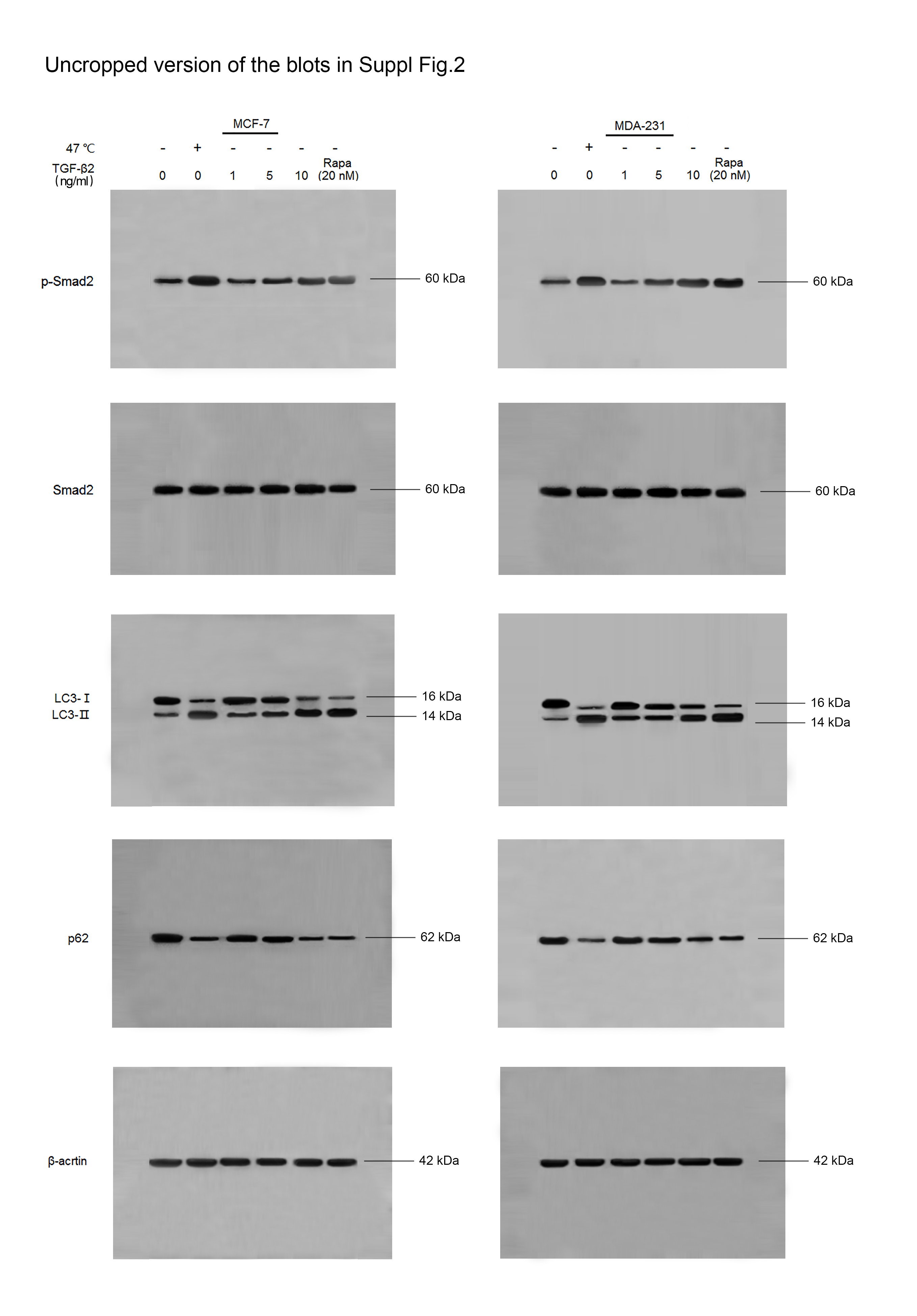

Supplement: Supplemental Information 6 — Raw data of Western blotting include Fig. 1–Fig. 3, Figs. 5 and 6. [file peerj-11-14640-s006.zip › Raw data of WB/Raw data of WB-TIF ╕±╩╜/Raw data of WB in supplementary Figures/Suppl figure 2.tif]

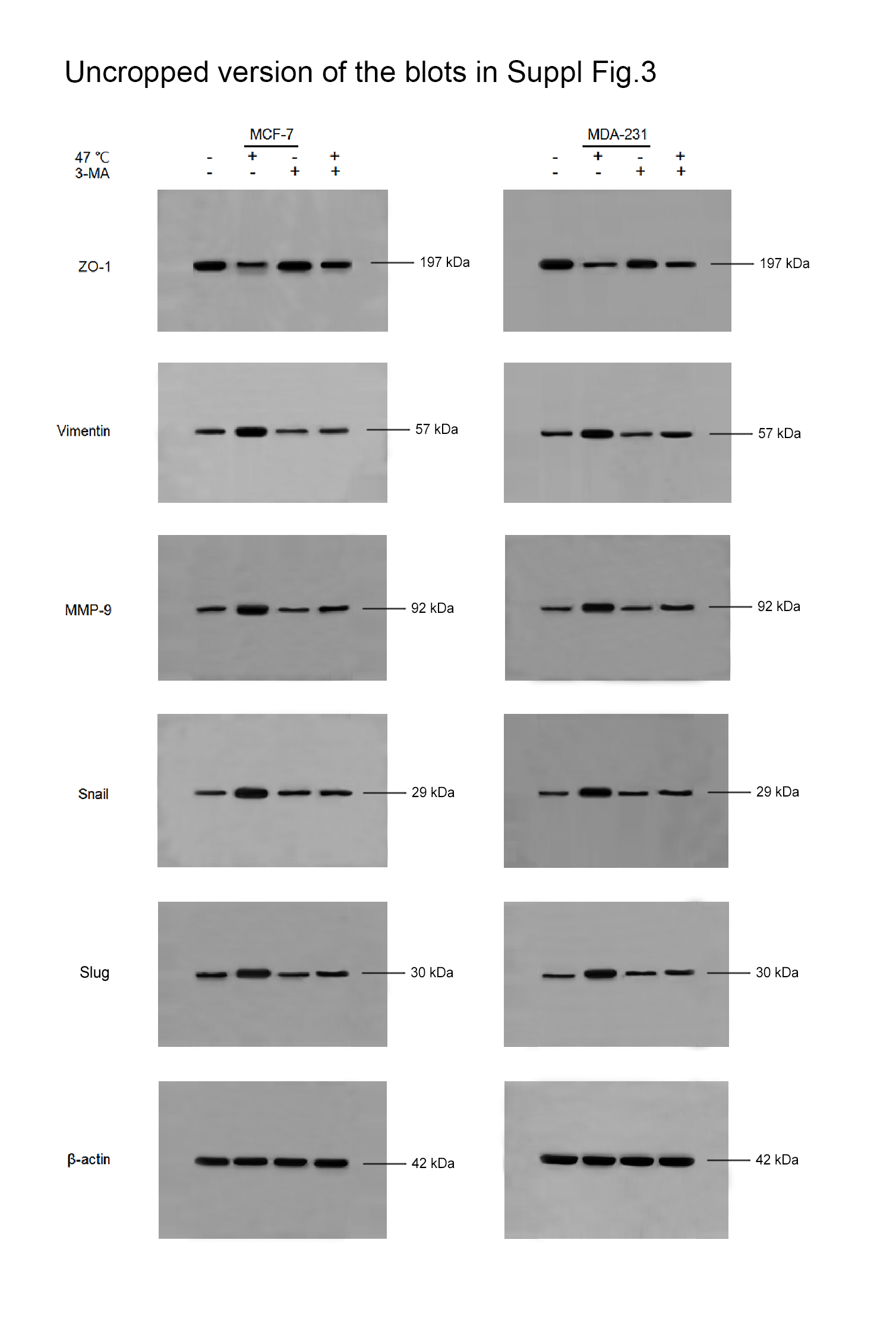

Supplement: Supplemental Information 6 — Raw data of Western blotting include Fig. 1–Fig. 3, Figs. 5 and 6. [file peerj-11-14640-s006.zip › Raw data of WB/Raw data of WB-TIF ╕±╩╜/Raw data of WB in supplementary Figures/Suppl Figure 3A.tif]
