# Supplementary material for: Heat treatment-induced autophagy promotes breast cancer cell invasion and metastasis via TGF-β2-mediated epithelial-mesenchymal transitions
Source: PeerJ. 2023 Jan 12;11:e14640. doi: 10.7717/peerj.14640 (PMC9840853; doi:10.7717/peerj.14640)
Supplement: Supplemental Information 4 [file peerj-11-14640-s004.doc]

**Table S1** Sequences of primers used in quantitative real-time PCR (qrt-PCR) analysis

| Gene | Forward primer (5’-3’) | Reverse primer (5’-3’) |
| --- | --- | --- |
| E-cadherin | ACCCAGGTCTTC TACTGC | GATGTGTTCTCGGTCCAGA |
| N-cadherin | CCTGAGGGATCAAAGCCTGGA | TTGGAGCCTGAGACACGATTC |
| α-catenin | GGCTTGAACCAACCTACGGATGAC | TCCTTCTGCCACTGTTCTCCTACG |
| β-catenin | CTGGTGAAATCCCGGAAAATGA | TTGCTGCCAAACTATCTTGTGA |
| Fibronectin | CTTACAACGTCAACGACACG | TGGGGTCACATTTCCATCTG |
| Vimentin | AGTCCGCACATTCGAGCAA | GGGGAAACCGTTAGACCAGAT |
| MMP-9 | CGAACTTTGACAGCGACAAGA | TCAGGGCGAGGACCATAGAG |
| SNAIL | CGAAAGGCCTTCAACTGCAAAT | ACTGGTACTTCTTGACATCTG |
| SLUG | CGCCTCCAAAAAGCCAAAC | CGGTAGTCCACACAGTGATG |
| ZO-1 | CACGCAGTTACGAGCAAG | TGAAGGTATCAGCGGAGG |
| GAPDH | GCTCTCTGCTCCTCCTGTTC | CGCCCAATACGACCAAATCC |
